# The relationship between the temporal structure of magnetoencephalography recorded brain activity and capacity to form discrete auditory representations

**DOI:** 10.1111/ejn.14289

**Published:** 2018-12-11

**Authors:** Christopher Allen

**Affiliations:** ^1^ School of Psychology Cardiff University Brain Research Imaging Centre (CUBRIC) Cardiff University Cardiff UK

**Keywords:** auditory, broad‐band, discrete representation, magnetoencephalography, oscillations

## Abstract

A function of oscillatory brain activity may be to align activity relative to threshold potentials and in doing so provide limited opportunities for representational neuronal assemblies to form. This low‐level function could apply across frequency bands and potentially affect the temporal dynamics of experience. To test these possibilities, a magnetoencephalography protocol was developed where capacity to form discrete auditory representations over time was assessed relative to oscillatory brain activity. Three sets of preregistered analyses were conducted. First, the capacity to form representations correlated with the prevalence and durations of activity localised to the auditory cortex. Second, brain oscillations became entrained to stimuli over a broad range of frequencies. Finally, a sequence of gamma (*γ*) band events predicted successful discrete representation, where previous research had indicated similar individuation‐related differences within the alpha (*α*) range. Together, these findings indicate that a low‐level function of cortical oscillations, which may apply across a range of frequency bands, is periodically to set conditions in which representational neuronal assemblies can manifest, limiting and so affecting the flow of experience.

**Abbreviations**
ANCOVA: analysis of covariance
ANOVA: analysis of variance
BF: Bayes factor
MEG: magnetoencephalography
PLV: phase locking value
SAM: Synthetic Aperture Magnetometry

## 1 INTRODUCTION

Field potential activity, as recorded via electro/magneto‐encephalography (E/MEG), is dominated by regularities which are understood as oscillations. The role played by oscillatory brain activity is commonly understood as consisting of narrow, frequency band‐specific functions. However, there are many inconsistencies in findings pertaining to frequency specificity and closely related functional properties have been linked to a broad range of oscillatory frequency bands (Pfurtscheller, Neuper, Pichler‐Zalaudek, Edlinger, & Lopes da Silva, 2000; VanRullen, 2016). While narrow frequency bands do perform task and modality specific functions, it is also possible that these are supported by a broadband, low‐level function. There may be intrinsic properties of oscillations, common across a range of frequencies, which lend themselves to, and enable, more frequency‐specific functions. One such property of oscillations is that they align activity, over time, relative to thresholds for action potentials, and, in doing so, can offer time‐limited conditions that are opportunities for the manifestation of representational neuronal assemblies (Buzsaki & Draguhn, 2004; Varela, Lachaux, Rodriguez, & Martinerie, 2001). In doing so, brain oscillations may limit how quickly one perceptual object can form and be separated from the next according to innate oscillatory rates. This proposed role could underlie a range of functional phenomena and even affect the flow of experience, which has testable implications.

The temporal structure of lived experience appears to be limited, where the minimum duration of individuated thoughts is approximately 100 ms (Husserl, Churchill, & Heidegger, 1964; Stroud, 1956). This periodicity is coincident with the most prevalent brain oscillatory rate in the *α* range (~10 Hz). This connection between durations available for individuated representations and brain oscillations was supported by early observations, recently replicated, in which closely presented pairs of visual stimuli either fuse or are discretely represented depending on when they are presented relative to the phase of ongoing occipital *α* oscillations (Milton & Pleydell‐Pearce, 2016; Varela, Toro, John, & Schwartz, 1981). This suggests that oscillations may determine when and how quickly discrete representations can occur. A range of studies in the visual (Busch, Dubois, & VanRullen, 2009; VanRullen, Reddy, & Koch, 2006) and somatosensory domains (Baumgarten, Schnitzler, & Lange, 2015) have continued to link specific frequency bands to capacity to parse representations over time. The continuous wagon wheel illusion has provided some of the clearest evidence for this, where illusory motion and stability of a spinning black and white disk arise from discrete parsing of stimuli (VanRullen, Zoefel, & Ilhan, 2014). Not only has the temporal frequency of the stimuli at which the illusion arises been shown to correspond cortical oscillations in the *α*/*β* frequency ranges (~10–20 Hz, Simpson, Shahani, & Manahilov, 2005; VanRullen, Reddy, & Koch, 2005) but the amplitude of occipital *α* oscillations has been shown to predict subjective transitions between parsed and merged percepts (VanRullen et al., 2006). However, it has also been reported that attempts to demonstrate similar relationships in audition have met with limited success (VanRullen et al., 2014). While direct evidence involving auditory tasks designed to probe discrete representation over time appears to be lacking, several studies have described oscillations entraining to auditory stimuli (e.g., Lakatos et al., 2013) and this entrainment, within the theta (*θ* ~5 Hz) range, has been linked to intelligibility of speech stimuli (Peelle, Gross, & Davis, 2013). While these observations are commonly conceived of as due to frequency band‐specific properties, they may also tap into this more fundamental property in the provision of opportunities for representation.

If oscillations provide time‐limited opportunities for representations, then tasks that probe the limits of capacity to represent over time should bear relationships to oscillatory activity. Furthermore, if this is a fundamental property of oscillations, such relationships should be observable across sensory modalities. This study aimed to probe this by quantifying capacity to form discrete auditory percepts over time and analysing their relationships to MEG recorded oscillatory activity. This motivated the development of an MEG task designed to push auditory discrete representational capacity to its temporal limits in a way that could be related to cortical oscillatory activity. Three sets of hypotheses were preregistered with the open science framework (see https://osf.io/h3z5n/) and found support in the data.

## 2 MATERIALS AND METHODS

### 2.1 Procedure

Methods were registered prior to data collection (https://osf.io/h3z5n/). Example stimuli, secondary exploratory analyses, including posttask monitoring are described in Supporting Information. Data, analysis and stimuli presentation code is available at https://osf.io/34rtm/.

The Research Ethics Committee at Cardiff University, School of Psychology, approved all procedures and all participants confirmed their written informed consent to participate, conforming to the standards of World Medical Association Declaration of Helsinki. Twenty participants completed the experiment (mean age 24.85 ± 5.55 *SD*, 14 female). One participant was excluded according to preregistered criterion (Supporting Information Appendix S5) for excessive head movement and self‐reported excessive fatigue (not included in the 20).

**Figure 1:**
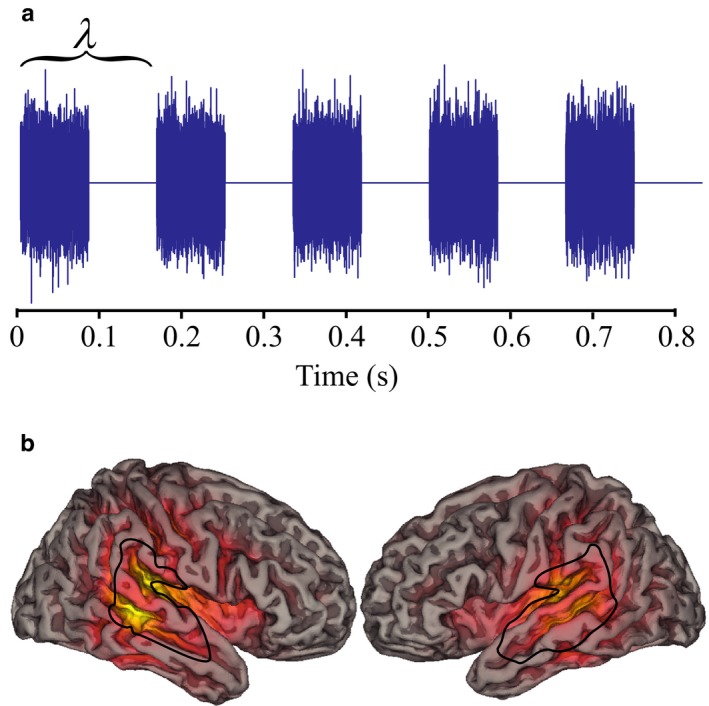
(a) Illustration of an example burst train presented on a single trial. *λ* refers to the wavelength of a burst cycle. In this example of five bursts are represented at 6 Hz. The task involved counting the number of bursts where between 4 and 7 bursts were presented over a range of frequencies (3–38 Hz). (b) Representation of spatial distribution of group‐evoked response from the auditory cortex where brighter yellow colours indicate increased magnitude and the black line surrounds the location of the auditory cortex from which data were drawn. [Colour figure can be viewed at wileyonlinelibrary.com]

The task involved presenting participants with trains of white noise bursts over a range of frequencies or rates (3, 4, 6, 8, 10, 12, 14, 16, 18, 22, 28 and 38 Hz). There were 4–7 busts within a train and the task was to count the number of bursts (Figure 1a, https://osf.io/jz54p/). The auditory burst stimuli were designed to track the mechanisms of discrete representation: When discrete representation failed the white noise of the stimuli should merge resulting in incorrect task performance.

Stimuli were produced using Matlab (Mathworks Inc, version 8.1.0.604) with the Psychophysics Toolbox (Brainard, 1997, version 3.0.11) and transmitted to the magnetically shielded room via E‐A‐Rtone (gold) insert earphones. Responses were collected via a Lumitouch (Photon Control) response box, where the four buttons on the upper surface corresponded to response options 4–7 from left to right. The noise bursts consisted of white noise of random amplitude fluctuation at each sample time with sample rate of 48,000 Hz (unfiltered, range [amplitude] adjusted according to participants preference, prior to the experiment).

Participants completed four blocks of 144 trials with short breaks (~1–2 min) in‐between blocks. Within each block, all 12 frequencies were presented at least 8 times, with equal prevalence of each burst condition. Two frequencies (10 and 14 Hz) were presented 32 times per block (see Section 2.3.3). Data were concatenated across all experimental blocks. Therefore, each frequency was presented at least 32 times and the higher prevalence frequencies (see Section 2.3.3) were presented 128 times. Trial orders were randomised. Trials were separated by a 1.25 second interval following the nonspeeded response. Prior to data collection, participants were familiarised with the task.

### 2.2 Data acquisition and preprocessing

MEG data were acquired on a 275 channel CTF system, sampling at 1,200 Hz in the supine position and analysed as third‐order synthetic gradiometers (Vrba & Robinson, 2001). Data were epoched into −800 to 800 ms trials, relative to trigger stimulus onset. This meant that a later portion of the full burst trains was not being captured for some of the lower frequencies of presentation, when trains contain higher numbers of bursts. The reason for this was to allow for consistent temporal structure of trials without introducing long intervals between trials at higher frequencies with lower numbers of bursts, as this had the potential to impact upon performance and data quality. This defined all trials without the inclusion of multiple trials into a single epoch. Trials were visually inspected, with the experimenter blind to trial condition, and clearly corrupted trials (e.g., by movement) were excluded (781 out of 11,520, 6.8%).

The peak‐evoked change was used to construct an auditory “virtual sensor” using Synthetic Aperture Magnetometry (SAM; Vrba & Robinson, 2001) constrained to the auditory cortex with a binary mask derived from the Harvard‐Oxford atlas (Desikan et al., 2006) representation of the superior temporal gyrus (anterior and posterior sections), thresholded to 25% (Abrams et al., 2011; see Figure 1b). The peak activation during a 50–400 ms active period following stimulus onset, over all trials, was isolated for each participant using mri3dX (Singh KD) and used to produce activity traces. The baseline applied comprised the final 350 ms prior to stimulus onset. The evoked SAM made use of a 200 Hz low pass filter. A 1 Hz high pass filter was also registered, but this was never applied to avoid suppression of evoked responses of interest. Also, in addition to registered methods, all analyses using baseline or active periods applied a +30 ms offset to timings to accommodate for transmission from the E‐A‐Rtone modals to ear inserts (~1 m).

All spectral oscillatory amplitude and phase information was estimated using a Hilbert transform applied to the resultant virtual sensor between 3 and 100 Hz with 1 Hz step size and 6 Hz bandwidth. All bandpass filters were bidirectional zero‐phase Butterworth filters. Unless otherwise stated (Section 2.3.2), these temporal parameters were used for all analyses.

### 2.3 Analyses

#### 2.3.1 Correlation between oscillatory activity and behaviour

As counting requires discrete representation, this task allowed investigation of the limitations of representational capacity and its correspondence to oscillatory activity. The behavioural measure was the normalised proportion correct at each frequency of presentation. At each of these data points, the normalised mean amplitude of the Hilbert envelope, across the active period, at the corresponding cortical frequency was the primary MEG‐dependent measure. In clarification of the preregistration document, both behavioural and amplitude measures were normalised for each participant by subtraction of the minimum value and division by their range and no prestimulus baseline was applied to the MEG measure.

Analysis of covariance (ANCOVA) was applied to assess the correspondence between the behavioural and spectral data, while treating participants as a partialled out categorical variable (Bland & Altman, 1995). The corresponding Bayes factor (BF) was the result of applying the JZS prior with default scaling (√0.5) to the *t*‐statistic of the regression slope (Rouder, Speckman, Sun, Morey, & Iverson, 2009). This Bayesian test was used for all *t*‐test equivalents. Secondary coefficients were used to further characterise relationships between measures. A one‐to‐one correspondence between individuation and oscillatory activity was predicted to result in the correlations intercept approximating to zero (BF < 1/3). Conversely, if more than one oscillatory cycle is required to individuate a percept, then the behavioural intercept coefficient, should be positive (BF > 3) and if less than an oscillatory cycle is required, the intercept coefficient should be negative, potentially falsifying the theory (BF > 3; Rouder et al., 2009). A range of individual differences‐related analyses are described in Supporting Information Appendix S1.

If brain oscillations provide conditions for representation, then separate oscillatory cycles may be required to discretely represent rapidly successive stimuli (Varela et al., 1981). The capacity to represent should be limited by, and track the presence of, oscillatory activity over a range of frequencies. The first hypothesis was therefore that task performance should correlate with the prevalence of oscillatory activity, expressed as amplitude spectra, over a range of frequencies.

#### 2.3.2 Entrainment of cortical response

The second series of analyses posed the question of whether oscillations were entrained to the stimuli, resulting in an amplitude modulation at the frequencies at which the stimuli were presented (Gross et al., 2013; Herrmann, 2001). Point values for oscillatory amplitude of the induced response, at each frequency of presentation were derived (matched). This applied a prestimulus baseline and took the mean across the active period at the frequency of presentation resulting in a single point value estimate of induced response. These values, therefore concern the induced oscillatory change in response to the stimuli, as opposed to the prevalence of oscillatory activity across a range of frequencies as applied in the first set of analyses. These values were compared to point values at the same oscillatory frequency, but where MEG data was drawn from all other frequencies of presentation (unmatched). Data were randomly down‐sampled so that trial numbers were equal across the comparison (see Supporting Information Appendix S2).

**Figure 2:**
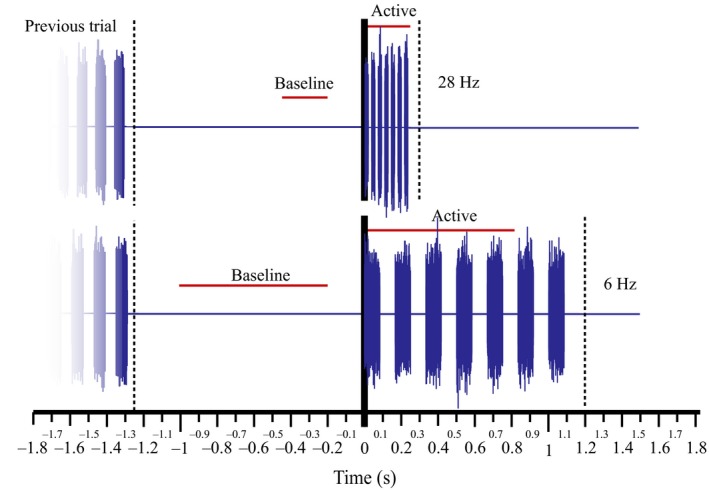
Illustration of temporal division of data applied in analysis of the effect of the stimuli upon oscillatory cortical response. Two trials are depicted where stimuli are presented at 28 and 6 Hz. Dotted lines indicate separate trials. The temporal structure differed from that previously described Section (2.2) in order to target the response across the course of the stimuli train, which differed according to frequency of presentation. [Colour figure can be viewed at wileyonlinelibrary.com]

As the question here relates to brain responses across the course of the train of stimuli, the temporal structure of the data differed from that described for other analyses. The active period applied here was from the onset of the first burst until 200 ms after the end of the final burst within a seven burst train. The baseline period covered an equal period, prior to stimuli onset, offset by 50 ms (see Figure 2). These periods were also limited to no more than 800 ms of data.

A repeated measures ANOVA was applied with factors of matched vs unmatched (two levels) probing the primary question of cortical entrainment by stimuli frequency, and the frequency of presentation (12 levels). A statistical interaction involving the frequency of presentation factor would suggest frequency‐specificity of the response. Chauvenet's criterion (Chauvenet, 1863) was applied to the main matched vs. unmatched comparison with a default criterion level of 0.5, resulting in the exclusion of one participant's data from the entrainment analysis.

The second main hypothesis predicted that brain oscillations, in performing their proposed role in providing opportunities for representation, should be affected by and become entrained to, stimuli (Gross et al., 2013; Lakatos, Karmos, Mehta, Ulbert, & Schroeder, 2008). This would express as a relative increase in oscillatory amplitude at the frequency of the stimuli.

#### 2.3.3 Successful vs. unsuccessful contrast

If brain oscillations fulfil their proposed role in representation then oscillatory state may reflect successful discrete counting. These analyses targeted three interrelated aspects: oscillatory amplitude, phase constancy and phase angle.

Two data sets were derived from the higher prevalence trials (10 and 14 Hz), for each participant, comprising successfully and unsuccessfully counted trials. Randomised down‐sampling was applied once so that for each participant an equal number of trials contributed to each data set (mean 90.9 ± 21.4 *SD*).

##### Oscillatory amplitude

The contrast between successful and unsuccessful counting was applied to oscillatory amplitude data with and without the baseline applied. This involved *t*‐tests with Chauvenet's correction (Chauvenet, 1863) at each frequency and time point from −800 to 800 ms with a frequency range of 3:100 Hz (1 Hz step size), using the variance across participants and mass cluster permutation correction (Groppe, Urbach, & Kutas, 2011; Maris & Oostenveld, 2007; Nichols & Holmes, 2002). Maximum cluster mass's were identified via the summed *t*‐statistics applying 8‐way connectivity over time and frequency (criteria for inclusion: *p* < 0.05) and 5,000 permutations.

Here, the prediction was that if an oscillatory cycle is required to successfully represent information then, because an oscillation will have occurred, oscillatory amplitude should be higher on successful trials compared to unsuccessful trials. Also, because the absence of such cycles prior to stimuli might be conducive to successful performance, a relative desynchronisation prior to, or coincided with, stimuli might be observed, in the *α* range (Busch et al., 2009; Rodriguez et al., 1999).

##### Phase consistency

The question posed here was whether there was consistency in the oscillatory phase angle when trials were successfully discretely represented, when they were not, and if there was any difference in consistency between these two conditions. Rayleigh tests were applied to successful and unsuccessful data sets separately (Zar, 2014) assessing phase consistency (H1) relative to uniformity (H0), using the circular mean phase angle across trials at the group level (Zar, 2014). The difference in consistency between conditions was assessed through comparison of phase‐locking values (PLV, see Lachaux, Rodriguez, Martinerie, & Varela, 1999), note here the PLV was computed across trials rather than sites and the Hilbert transform was used, as opposed to a complex Gabor wavelet, which can be described as follows:

$${\text{PLV}}_{t}=\frac{1}{N}\sum_{n=1}^{N}e^{\left(\int \theta (t,n)\right)}$$

where *t* are the time points, *n* are the trials or group (*n*[1,…,*N*]) and *θ*(*t*,*n*) is the angular difference across sets of trials (*ϕ*1–…*ϕn*). Hence, perfectly aligned phase angles across trials will have a PLV value of 1 and approach 0 as phase consistency is lost. A set of difference scores between successful, unsuccessful conditions were compiled based on PLV and *t*‐test related cluster statistics previously described were applied.

The hypotheses here were that phase may be more consistent for successful trials than unsuccessful, resulting in a positive PLV difference observed as a predictor of success shortly before the stimuli, in the lower frequency range (Busch et al., 2009).

##### Phase angle

These analyses attempted to test the difference in circular mean angular phase of oscillations, across trials, between the successful and unsuccessful conditions with a series of Watson–Williams tests (Zar, 2014). This test was replaced by nonparametric Watson's *U* ^2^ tests, owing to violations of homogeneity (Zar, 2014). This involved 5,000 permutations at the group level and the mass cluster correction used Wanton's *U* ^2^ test statistic.

The prediction here was that behavioural success may have been more prevalent when the onset of stimuli were coincident with the negative trough of oscillatory cycles within the lower frequency ranges (Romei et al., 2008; Varela et al., 1981).

## 3 RESULTS

### 3.1 Correlation

**Figure 3:**
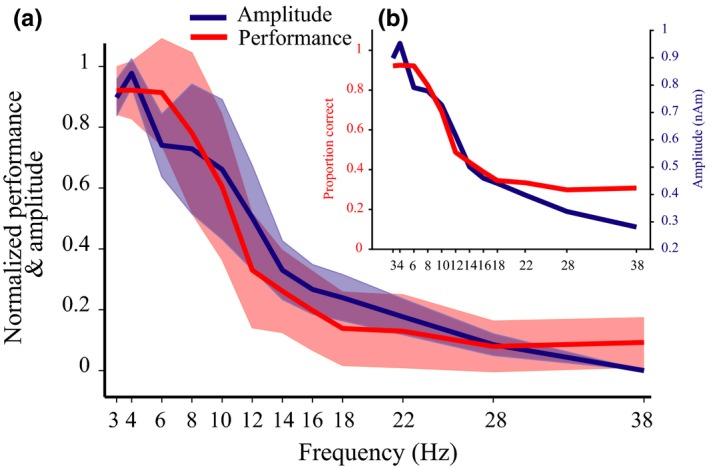
Group mean amplitude and behavioural performance against frequency (of presentation and oscillation). Oscillatory amplitude was that of the Hilbert envelope applied to a virtual sensor activity trace constructed at participants’ peak‐evoked location within the auditory cortex. Performance refers to participants’ capacity to count the numbers of bursts. (a) Illustration of normalized data to which analyses were applied. Normalisation of both measures involved subtraction of minimum levels from their raw value and division by their range. Shaded areas represent 1 *SD*. (b) Presents the same group mean data but in raw native units. [Colour figure can be viewed at wileyonlinelibrary.com]

Over the range of frequencies at which the stimuli presented raw behavioural performance, as proportion correct transitioned between 0.92 (±0.11 *SD*) at 4 Hz and 0.29 (±0.08 *SD*) at 28 Hz where chance performance is 0.25. Figure 3 illustrates the within‐subject correlation (Bland & Altman, 1995) observed between accurate counting performance and prevalence of oscillatory activity, over a range of frequencies (*r* = 0.88, *F*_1,219_ = 717.63, *p* = 4.79 × 10^−71^, BF = 2.81 × 10^13^) supporting the first hypothesis and demonstrating that they exhibit a corresponding structural profile. Some form of correlation is to be expected when both measures conform to a 1/frequency distribution as observed here. However, an additional aspect of the analysis was the prediction that, if an oscillatory cycle is required to individuate content, then the behavioural distribution should overlay the oscillatory distribution over the same range of frequencies, as plotted in Figure 3. Statistically, this consistency was summarised by the ANCOVA's intercept approximating to zero (−0.005[±0.021 *SE*], *T*_19_ = −0.22, *p* = 0.83, BF = 0.24), indicating a one‐to‐one relationship between capacity to represent over time and brain oscillations. Differences between participants’ individuation performance appeared to be related to their specific oscillatory make up, explored via the participant factor of the ANCOVA (*F*_19,219_ = 3.02, *p* = 4.76 × 10^−5^, BF = 6.92). However, this last comparison is exploratory and other individual difference‐related examinations where inconclusive (see Supporting Information Appendix S1), this suggests the possibility that differences in participants’ oscillatory brain activity may predict their specific ability to perform the task (Palva et al., 2013). Alternative post hoc explorations of oscillatory and behavioural relations are available in Supporting Information.

### 3.2 Entrainment

**Figure 4:**
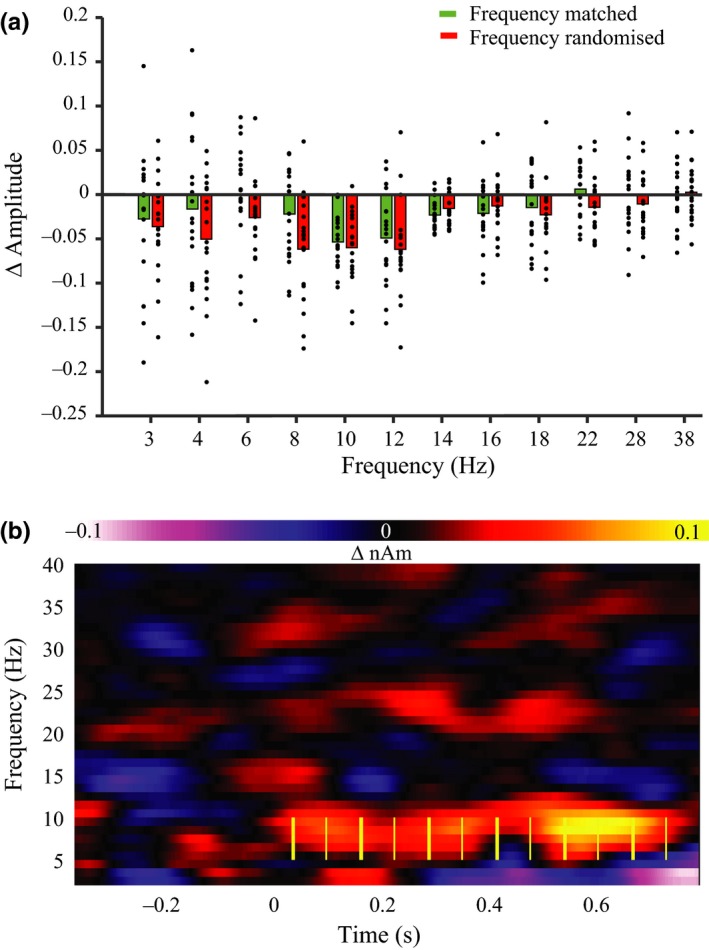
Entrainment analyses. (a) Mean (bar) and participants’ induced amplitude where data was drawn from either the matching frequency of presentation or where data was randomly downsampled from other nonmatching frequencies. (b) Example of group level time × frequency representations of differences described in a, yellow lines illustrate the stimuli (at 8 Hz in this example) where thicker lines represent burst onset and thinner that of silent periods. [Colour figure can be viewed at wileyonlinelibrary.com]

The second set of analyses supported the prediction of a relative increase in oscillatory amplitude at the frequencies at which the stimuli were presented (*F*_1,18_ = 10.22, *p* = 0.005, BF = 9.30, Figure 4). This effect was apparent over the range of frequencies tested (validity × frequency interaction *F*_11,198_ = 1.05, *p* = 0.40, BF = 0.38). These indicate that oscillatory brain activity entrained to the stimuli and did so over a range of frequency bands.

### 3.3 Success

**Figure 5:**
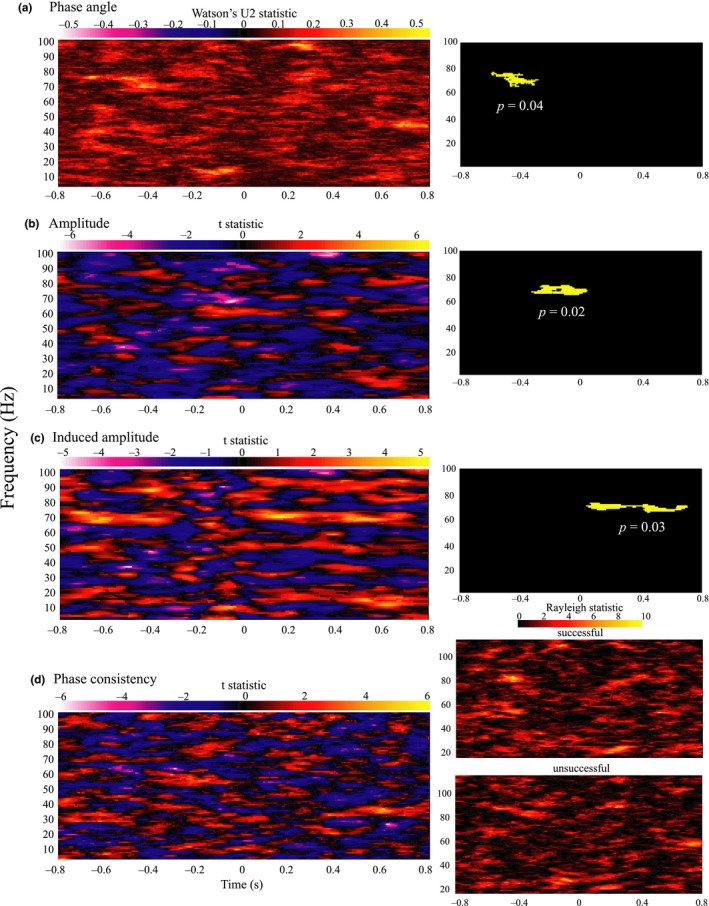
Time × frequency representations of group differences between successful and unsuccessful task performance in (a) phase angle (b) amplitude (c) amplitude with prestimulus baseline applied and (d) phase consistency (with phase consistency in each condition represented on right). Yellow on black panels indicate significant clusters. [Colour figure can be viewed at wileyonlinelibrary.com]

The final set of analyses contrasted successful vs. unsuccessful burst individuation, indicating a progression of *γ* range differences in both oscillatory amplitude and phase angle (Figure 5). First prestimulus phase angle differences occurred from −626 to −313 ms, at 66–77 Hz (angular difference of ~*π*, cluster *p* = 0.037). Next, amplitude desynchronisation in the successful condition was observed, predominantly prior to stimuli onset (66–73 Hz, from −356 to 18 ms, cluster *p* = 0.018), followed by a relative induced poststimulus synchronisation (66–73 Hz, 7–678 ms, cluster *p* = 0.027).

## 4 DISCUSSION

This study linked cortical temporal dynamics to formation of discrete auditory percepts. The correlational approach of the first analysis was preregistered with the aim of assessing and summarising coupling between oscillatory amplitude spectra and capacity to represent, over a range of frequencies. Overall, the rates over which the auditory cortex was observed to operate matched the rates at which participants were able to form discrete representations. The distribution of discrete representational capacity closely resembled that of innate activity, both conforming to a 1/frequency (*f*) pink noise structure. The ubiquity and functional significance of scale‐free 1/*f* is controversial (Farrell, Wagenmakers, & Ratcliff, 2006; Kello et al., 2010; Manning, Jacobs, Fried, & Kahana, 2009), and whether the observed spectral distribution refers to oscillations per se, as opposed to structured noise, is a matter of interpretation. At a minimum, the amplitude measure denoted underling activity with a periodicity that repeats at frequencies of central interest to this investigation, and, as repetitious, falls under a broad interpretation of the term “oscillatory”. The critical point is that the frequency range of the distributions are limited and, therefore, not scale‐free. Previous investigations of 1/*f* in brain and behaviour have found relationships over very different frequency ranges to those observed here (e.g., He, 2011; Van Orden, Holden, & Turvey, 2003). Instead the observed amplitude range corresponded directly to the range over which participants could perform the task of forming discrete representations. As cortical oscillatory rates are thought to be determined by the intrinsic size and conduction velocity properties of the brain (Buzsaki & Draguhn, 2004), representational capacity may be limited and determined by commonly observed oscillatory rates.

The current task pushed the auditory system to its limits in terms of how quickly participants were able to form discrete consecutive representations. This capacity corresponds to the prevalence of observable oscillatory activity Section (3.1) and the cortex responded, through entrainment, to the frequency‐specific characteristics of the stimuli Section (3.2). Both of these observations were made across a broad range of frequencies. Quantification of fine temporal resolution activity almost unavoidably involves decomposition in the frequency domain. To some extent, this entails a band‐specific understanding of oscillatory function. The success of demonstrations of functionally distinct oscillatory frequency bands (e.g., Lopes da Silva, 2005), together with the ambiguity in interpretation of spectral power (oscillatory vs. pink noise) are reasons for the dominance of a band‐specific perspective in neuroscience. Although less common, broadband relationships to behaviour, neural activity and experience are increasingly being recognised (Manning et al., 2009; Miller et al., 2014; Palva et al., 2013; Wen & Liu, 2016) and have even been applied where a priori band‐specific hypotheses failed to capture the data (Muthukumaraswamy et al., 2013). Narrow and broadband functionality are not mutually exclusive (Haller et al., 2018) and the observations made here support the proposal for a general low‐level function of the field potential activity in the alignment of activity, constraining and allowing representations, which may apply across a broad range of frequencies but is also likely to have frequency‐specific expressions.

The successful vs. unsuccessful contrast revealed a progression of events, specifically within the *γ* range, suggestive of a state difference conducive to rapidly forming discrete representations. Previous research has commonly associated *α* band changes with discrete representation (Busch et al., 2009; VanRullen et al., 2014). Therefore, the preregistered hypotheses specifically predicted *α* modulation, making the *γ* observations serendipitous. However, the synch/desynchronisation directionalities observed, relative to the onset of stimuli were predicted (https://osf.io/h3z5n/) and the succession over a range of measures across time supports its credibility as a physiological phenomenon of interest. Furthermore, broader‐band success‐related differences may be obscured by countermanding lower frequency changes (Manning et al., 2009). Additionally, the current observations are consistent with previous research linking prestimulus *γ* band fluctuations to states of preparedness or allocation of attention, particularly prior to demanding task elements, that allows for successful task performance (Reinhart, Mathalon, Roach, & Ford, 2011; Todorovic, van Ede, Maris, & de Lange, 2011; Womelsdorf, Fries, Mitra, & Desimone, 2006). The *γ* band finding, together with previous *α* band phenomena (Busch & VanRullen, 2010; Romei et al., 2008; Varela et al., 1981), indicates that closely related functional roles can be supported by a range of oscillatory frequencies, depending on context, and therefore highlights the contribution made by a range of oscillatory activity to the provision of conditions for representation. Speculatively, relatively fast *γ* cycles may optimise susceptibility by increasing the number of opportunities for representation over a given period. In such a manner, higher level functions may be fulfilled by narrow frequency bands, but are nonetheless dependent upon lower level functions, in the provision of conditions for representation.

This study proposed a role for aligned cortical activity in relation to the provision of opportunities for representation (Buzsaki & Draguhn, 2004). More specifically, for a neuron, or set of neurons, within a population to exert influence and contribute to representation, a specific pattern of conditions over time must obtain (Buzsaki & Draguhn, 2004; Hasselmo, Bodelon, & Wyble, 2002; Zohary, Shadlen, & Newsome, 1994). Alternating through periods of suppression and facilitation brings ionic potentials into temporal alignment relative to threshold for action potential. This temporal dynamic constrains potential outcomes (Friston, 2010), offers opportunities and sets conditions to enable specific patterning of activity. This can bring transient neuronal assemblies into relief, thus enabling and delineating representations (Buzsaki & Draguhn, 2004; Zohary et al., 1994). Transient formation of assemblies of this nature have been linked to the formation of conscious objects (Varela et al., 2001). The current findings relate cortical rates to the maximum rates of discrete representation, suggesting that the measured activity delimits the flow of conscious experience.

## CONFLICT OF INTEREST

None.

## Data Availability

Anonymized data and code is available at https://osf.io/h3z5n/. Please see the summary readme document.
