# Supplementary material for: The relationship between the temporal structure of magnetoencephalography recorded brain activity and capacity to form discrete auditory representations
Source: Eur J Neurosci. 2018 Dec 11;49(12):1564–74. doi: 10.1111/ejn.14289 (PMC6618102; doi:10.1111/ejn.14289)
Supplement: Supplementary file 1 [file EJN-49-1564-s001.docx]

Supporting Information Appendix: The relationship between the temporal structure of magnetoencephalography recorded brain activity and capacity to form discrete auditory representations.

This section supplements the main text describing exploratory analyses and alternative representations of the data. It follows the structure of the analyses in the pre-registration document and main text, consisting of sections pertaining to: 1) the relationship between oscillatory and behavioural distributions, 2) the entrainment of oscillations by stimuli and 3) the divisions of data according to behavioural success. These are then followed by a description of the analyses of the questionnaire data (4), exclusion criteria (5) and participant instructions (6). Anonymised data and analysis programs from this experiment are available athttps://osf.io/34rtm/ and example stimuli are available at <https://osf.io/jz54p/>.

**S1. Correlation between oscillatory activity and behavior**

An alternative graphical representation of the primary ANCOVA relating the oscillatory and behavioral data is presented in figures SI1 and SI2 (Bland & Altman, 1995a). Figure SI1-2 use the same data as figure 3 of the main text but provides a more complete, although less intuitively interpretable, representation of the data

An alternative exploratory statistic which summarizes the correspondence between the oscillatory and behavioral data is the interclass correlation coefficient (Shrout & Fleiss, 1979) applied to the ANCOVA which was 0.956.

The primary analyses of this section investigated the relationships between behavioral performance and the raw oscillatory amplitude quantified via the application of a Hilbert envelope, without pre-stimulus bassline, linking the measures to the presence of oscillatory activity rather than the cortical response. Additionally, the same main analyses were applied but where a pre-stimulus baseline was subtracted in order to test the induced oscillatory response to the onset of the stimuli. In this exploratory analysis the general partnering of the data, together with the strong correspondence between the oscillatory and behavioral distributions, was maintained, although the effect size was slightly reduced (r=0.56, F_(1,219)_=100.60, p=1.01×10^-19^, BF=2.38×10^6^). Reduction in effect magnitude can be expected following the subtraction of the baseline which itself contains random fluctuations. The oscillatory distribution of this induced change from pre-stimulus levels was now to the left of the behavioral distribution (see figure SI4) and the resultant intercept of the ANCOVA in this analysis was significantly positive in the behavioral direction (value=0.219(± 0.031SE), T_(19)_=7.16, p=1.24×10^-11^, BF=2.10×10^4^). An additional ANOVA of residual error was described in the pre-registration document. This was aimed at exploration of any discrepancy between the measures over the frequencies under investigation. However, as the primary analysis of the intercept supported the absence of their bringing a discrepancy, the corresponding ANOVA was redundant and therefore not applied.

Whether differences in individual participants’ performance of the task corresponded to differences in their oscillatory brain activity was investigated in number of ways. This question of individual differences in behavioral performance predicting individuals oscillatory make up was explored most succinctly, directly and utilizing more of the data, via the participant factor of the ANCOVA reported in the main text. Although this last comparison is a post-hoc exploratory analysis, it suggests that differences in participants’ oscillatory brain activity may predict their specific ability to perform the task. However, this question was also posed by collapsing the behavioural and spectral data across frequencies and testing the correlation between the two means, across participants (Bland & Altman, 1995b), using a Pearson’s correlation and complimentary Bayesian correlation (Wetzels & Wagenmakers, 2012). This question was additionally posed at each frequency of interest. No correlation was observed using the mean performance and amplitude, across frequencies (r_(18)_=0.19, p=0.42, BF=0.24) or within any of the frequencies tested (see table SI1). A one sampled T-test applied to the resultant correlation coefficients, also had the capacity to demonstrate an individual difference-based relationship but did not support such a difference (T_(11)_=1.04, p=0.32, BF=0.45).

Following registration of this experiment and data collection it also became apparent that the 1/frequency distribution observed in both the behavioral and oscillatory data can be summarized by a single coefficient β within the formulation: Amplitude or Behavior ≈1/frequency^β^ (Palva *et al.*, 2013; He, 2014). 1/frequency curves were therefore fitted to the group mean oscillatory and behavioral data, using Matlab's curve fitting toolbox with default randomized initial fitting parameters. The similarity or differences between oscillatory and behavioral β coefficient were then assessed with a paired t-test and Bayesian equivalent (Rouder *et al.*, 2009). Additionally, as an exploratory analysis based on previous research (Palva *et al.*, 2013), individual participants’ behavioral and oscillatory β coefficients were correlated with one another. The resultant group mean β coefficient from the model fits to the behavioral data was (±95% confidence bounds) 0.35(0.16) where r^2^_(11)_= 0.52 and for the oscillatory data β was 0.33(0.13) and r^2^_(11)_= 0.56. The tests applied indicated the β coefficients’ numerical proximity across the measures (T_(19)_=1.03, p=0.32, BF=0.37). These β values correspond closely to previous reports (Linkenkaer-Hansen *et al.*, 2001). The proximity of the behavioral and oscillatory β values and overlap in the confidence interval is consistent with the overall prediction and interpretation that there is a relationship between the expression of oscillatory activity and behavioral capacity to individuate information over a range of frequencies and that the distribution of the two express a similar structure. The correlation of β values, resulting from individual participants’ fits, between the two measures (Palva *et al.*, 2013), but did not support the presence of such a relationship between β coefficients: r^2^_(18)_= 0.16,p= 0.50, BF= 0.21.


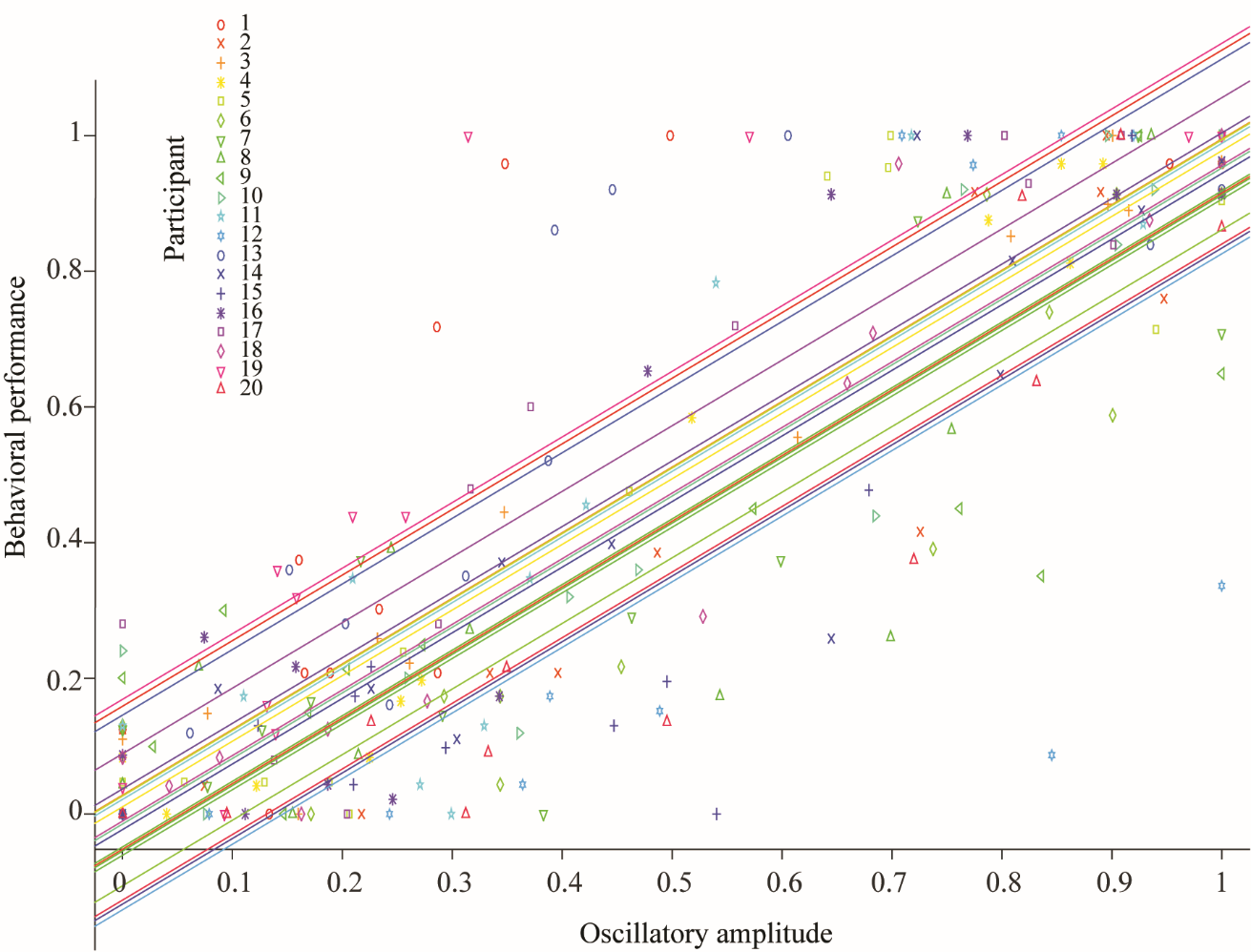


**Figure SI1.** Normalized behavioral performance in burst counting against normalized oscillatory amplitude taken from the auditory cortex. Parallel lines fitted for each participant illustrating the ANCOVA (Bland & Altman, 1995a).


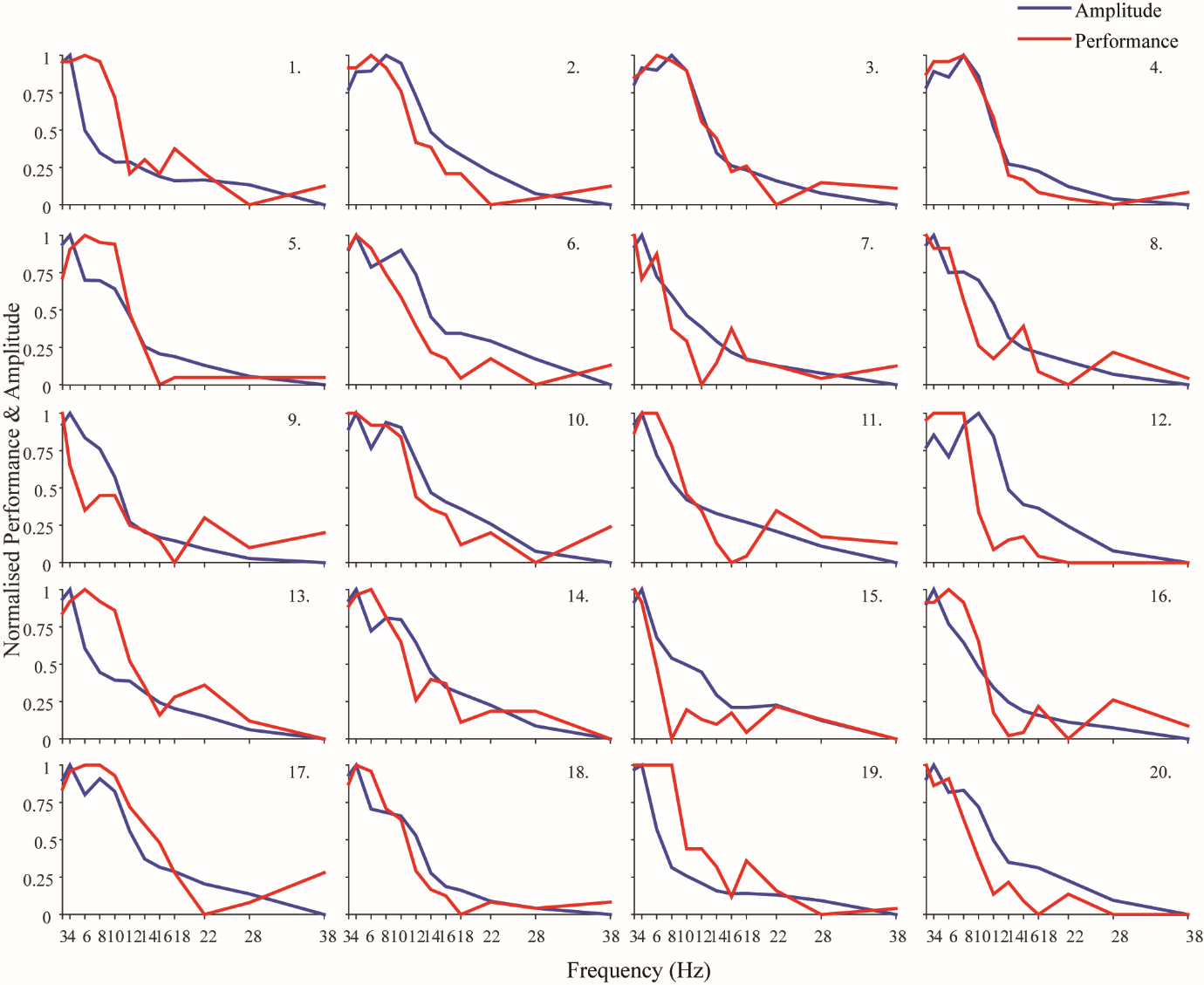


**Figure SI2.** Normalized behavioral performance and normalized oscillatory amplitude for individual participants. These are the individual participants’ distributions that contributed to figure 3.

**
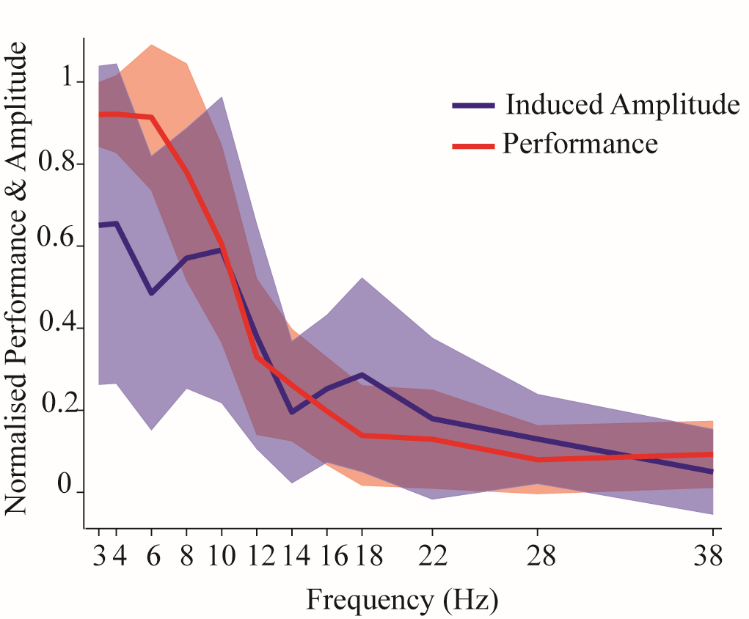
**

**Figure SI3.** Group mean induced amplitude and behavioral performance against frequency (of presentation and oscillatory). This figure conforms to the same structure as figure 3 of the main text but here a pre-stimulus baseline was subtracted to express the distribution of oscillatory activity in response to the stimuli.


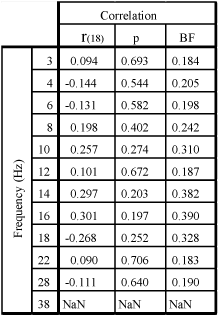


**Table SI1.** Correlation coefficient (r_(df)_) and corresponding Bayes Factor (Wetzels & Wagenmakers, 2012) for correlation between behavioral performance and oscillatory amplitude at each frequency are reported. Tests were not applicable at 38Hz as corresponding normalize oscillatory values where zero.

**S2. Entrainment of oscillations by stimuli**

The entrainment effect was tested with an ANOVA which compared the amplitude of the Hilbert envelope between where data was drawn at the frequency at which the stimuli were presented and compared this to the same amplitude measure but where data was drawn from other frequencies of presentation. In addition to the main effect of matched vs. unmatched and its interaction with frequency, reported in the main text, there was evidence for a main effect of frequency (F_(11,198)_=5.42, p=1.63×10^-7^, BF=2.02), which is in line with the first set of analyses. In accordance with pre-registered criteria, the numbers of trials turned out to be insufficient to probe the potential interaction between the entrainment effect and behavioral success, so this possibility remains an intriguing avenue for future research.

Following registration a discrepancy of interpretation between frequentist and Bayesian ANOVA and t-tests was noted which requires clarification. Under a frequentist approach the simple main effect of a two level factor (e.g. matched vs. unmatched) of an ANOVA is equivalent to a t-test applied to the means across other conditions, where F=√T. This is not the case for default Bayesian ANOVAs (Rouder *et al.*, 2012), where differences within, or the presence of, factors that are not of interest to the primary question, effects the outcome of the primary simple main effect analysis. This makes interpretation difficult. Therefore the BFs reported in the main text are the result of applying the Bayesian t-test to mean differences between conditions where there is no inconsistency in interpretation (Rouder *et al.*, 2009; Dienes, 2014) and these are supplemented for completeness by the results of the Bayesian ANOVA (Rouder *et al.*, 2012) as follows: the main effect of matched vs. unmatched computed as BF for main effects over the BF for the frequency main effect was 3.51. The interaction (full model over the main effects model) was 0.038 and the frequency main effect (main effects over validity main effect) was 3.90×10^6^. The results of the two approaches were therefore broadly consistent and differences concerned the relative weight of evidence.

In the interest of transparency the following additions to the registered procedure are noted. Initial inspection of the data indicated a significant effect of matched vs. unmatached (F_(1,19)_=12.88, p=0.002, BF=20.45), however the temporal parameters used in this computation were incorrect by 30ms (the time allowed for stimuli to reach participants’ ears from the speakers had been omitted). Therefore, it was necessary to recomputed the analysis, the results of which are reported in the main text. In doing so it became apparent that the result was dependent on the registered randomized down-sampling applied in the construction of the invalid contrast data set. To minimize this issue an additional analysis was undertaken where all trials were used, the results of which are reported below as explicitly exploratory and are not susceptible to randomization effects. The resultant non down-sampled contrast made use of 536.95 ± SD 47.23 mean number of trials for each participant in the matched condition and 5906.45 ±SD 519.57 in the unmatched condition. This compares to the mean of 536.95 ± SD 47.23 trials in both conditions under the pre-registered down-sampling approach. This exploratory analysis, undertaken to ensure that observed effects where not the result of the down-sampling, involving utilization of all available data. This supported predictions that stimuli entrained brain activity, involving relatively increased oscillatory amplitude at the frequency at which the stimuli were presented (F_(1,19)_=10.90, p=0.004, BF=11.69). Again no evidence for an interaction between validity and frequency was observed (F_(11,209)_=1.38, p=0.18, BF=0.42) and there was evidence for a main effect of frequency (F_(11,209)_=6.26 p=6.59×10^-9^, BF=2.72). The complimentary Bayesian ANOVA statistics, computed as above, are as follows: BF matched vs. unmatched =1.88, BF frequency=2.65×10^9^, BF interaction=0.058. Figure SI4 reflects the entrainment analysis applied were all trials where used as opposed to down-sampled data sets reported in figure 4 of the main text.

**
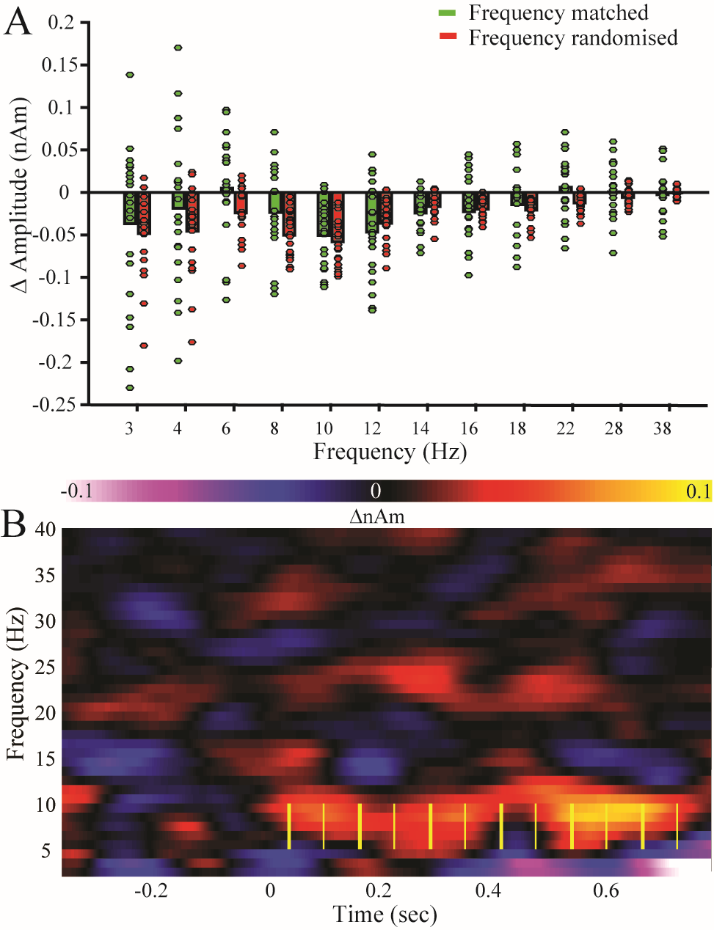
**

**Figure SI4.** Entrainment analyses. (**A**) Induced amplitude change, where data is drawn from either the matching frequency of presentation or *all* trials at other frequencies. Bars are of group mean and data points are individual participants’ data. (**B**) Example of group level time × frequency representations of differences described in a, yellow lines illustrate the stimuli (at 8Hz here, where thicker lines represent burst onset and thinner that of silent periods).

An additional exploratory analysis probed potential relationships between entrainment and behavioral performance, asking if the magnitude of entrainment correlated with individuals’ capacity to perform the task. This involved correlating participants behavioral performance with the difference between matched and unmatched induced amplitude, which the preceding registered analysis had suggested expressed entrainment. Using participant means across frequencies there appeared to be a weak trend (r= 0.35, p=0.13, BF_10_=1.49, BF_10_ were derived using JASP (0.8.1, applying Pearson’s rho correlation with a positive paired prior)) consistent with such a relationship, although there was little evidence of such a relationship at each of the frequencies analyzed (see table SI2). Therefore, no strong conclusions can be drawn with respect to these exploratory analyses.

**

**

**Table SI2**. Exploratory correlation between the entrainment effect and behavioral performance. The entrainment effect was quantified as the difference between by matched and unmatched amplitude measures and the performance measures was the normalised proportion correct. Presented are the correlation coefficients and corresponding p and BF_10_ values at each frequency and using the mean across frequencies.

**S3. Successful vs. unsuccessful contrast**

The pre-registration document described the potential of exploring a secondary, more restricted frequency set (3:18Hz, as opposed to the 3-100Hz described in them main text). However, this was redundant owing to the differences apparent within the primary analysis beyond the restricted range. Also as high prevalence data sets were merged, in order to meet the registered criteria of a minimum of 20 trials per condition, it was not possible to assess the interaction between behavioral success and frequency of presentation.

Supplementary to the cluster-based correction, a false discovery rate (FDR (Benjamini & Hochberg, 1995; Busch *et al.*, 2009)) correction was described in the pre-registration document and was applied to the p values derived from the time × frequency comparisons described. However, there are violations of the independence assumption of FDR correction, when applied to the time × frequency data. The option of an amendment to overcome this dependency (Benjamini & Yekutieli, 2001) did not seem appropriate as this would have penalized analyses for adjoined, correlated expressions of differences, which are theoretically more plausible than separate, independent differences. The violation of independence does however mean an emphasis should be placed on the supplementary and exploratory nature of the FDR-corrected tests. The application of FDR correction to time × frequency analyses applied at the group level revealed three significant clusters when applied to the unbaselined amplitude difference between successful and unsuccessful trials only (see figure SI5). These differences were again within the γ range: the first was at 37Hz and from 526 to -508ms relative to stimuli onset, the second was between 67 and 68Hz from -76 to -25ms corresponding to the time × frequency region highlighted by the mass cluster correction, and finally a 98 to 99Hz post-stimulus difference survived the FDR correction between 338 and 399ms.


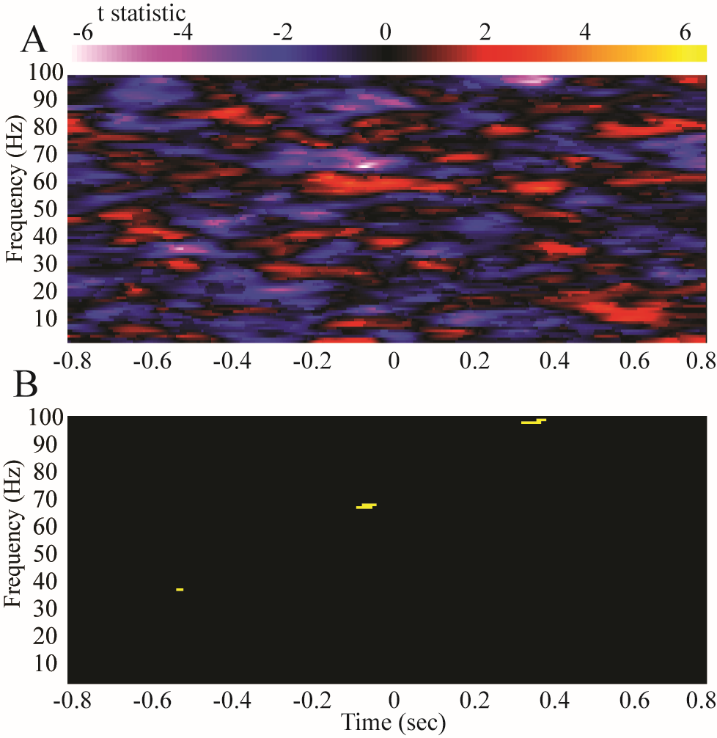


**Figure SI5.** (**A**) Time × frequency plot of t-statistic difference between successful and unsuccessful trials applied to the amplitude of the Hilbert envelope. (**B**) Depicts the regions of these differences which surpassed false discovery rate correction for multiple comparisons (Benjamini & Hochberg, 1995).

It is possible that similar randomization effects as those reported with respect to the entrainment analysis could have affected these analyses. However, the use of all trials here, as used in the entrainment analysis to alleviate this concern, would have caused substantive violations to the assumptions of the majority registered analyses of this section, particularly those that involved permutations of trial conditions (i.e. Watson’s U2 static and mass cluster based corrections) and those involving quantification of phase locking. Of the tests comparing successful and unsuccessful trials, this left only the amplitude and PLV t-static based difference, where FDR correction for multiple comparisons was applied, as the set of tests which could be applied and utilized all trials. These indicated a similar patterning to the main registered analyses, with a significant γ band pre-stimulus desynchronisation which survived FDR correction for multiple comparisons. As depicted in figure SI6 the FDR correction indicated that this occurred between -302 to -251ms and between 50 and 51Hz. Additionally, there appeared to be a brief post stimulus difference at 55Hz from 465 to 473ms.


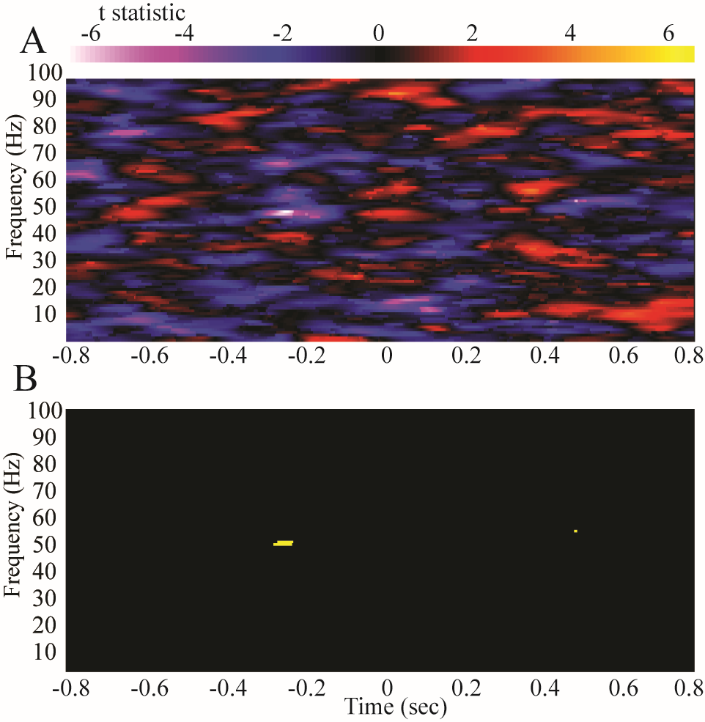


**Figure SI6.** (**A**) Time × frequency plot of t-statistic difference between successful and unsuccessful trials applied to the amplitude of the Hilbert envelope where the data to which analysis were applied consisted of *all* available trials rather than the downsampled balanced subset. (**B**) Depicts the regions of these differences which surpassed false discovery rate correction for multiple comparisons (Benjamini & Hochberg, 1995).

Individual participants’ data were also analyzed in a secondary set of analyses where variance was drawn across trials, rather than participants. At the individual participant level a deviation from the pre-registered protocol was applied, reducing the numbers of permutations to 1000, due to computational demands, of the phase analyses in particular. The effect of this reduction, if anything, is to make these analyses more conservative. At the participant level, the phase consistency analyses unlike the other measures, could not be computed as the measures used the variance across trials in their construction. Also it was not possible to apply the mass cluster-based correlation for multiple comparisons to the Rayleigh tests as these tests did not involve a direct comparison between successful and unsuccessful conditions. These two points are clarifications and additional to the original pre-registered document. A supplementary point value analysis described in the pre-registration document was not applicable here owing to the merging of high prevalence data sets. Figures SI7-11 are the time × frequency plots of these comparisons and the summary statistics based on the mass cluster based approach are summarized in table SI3.


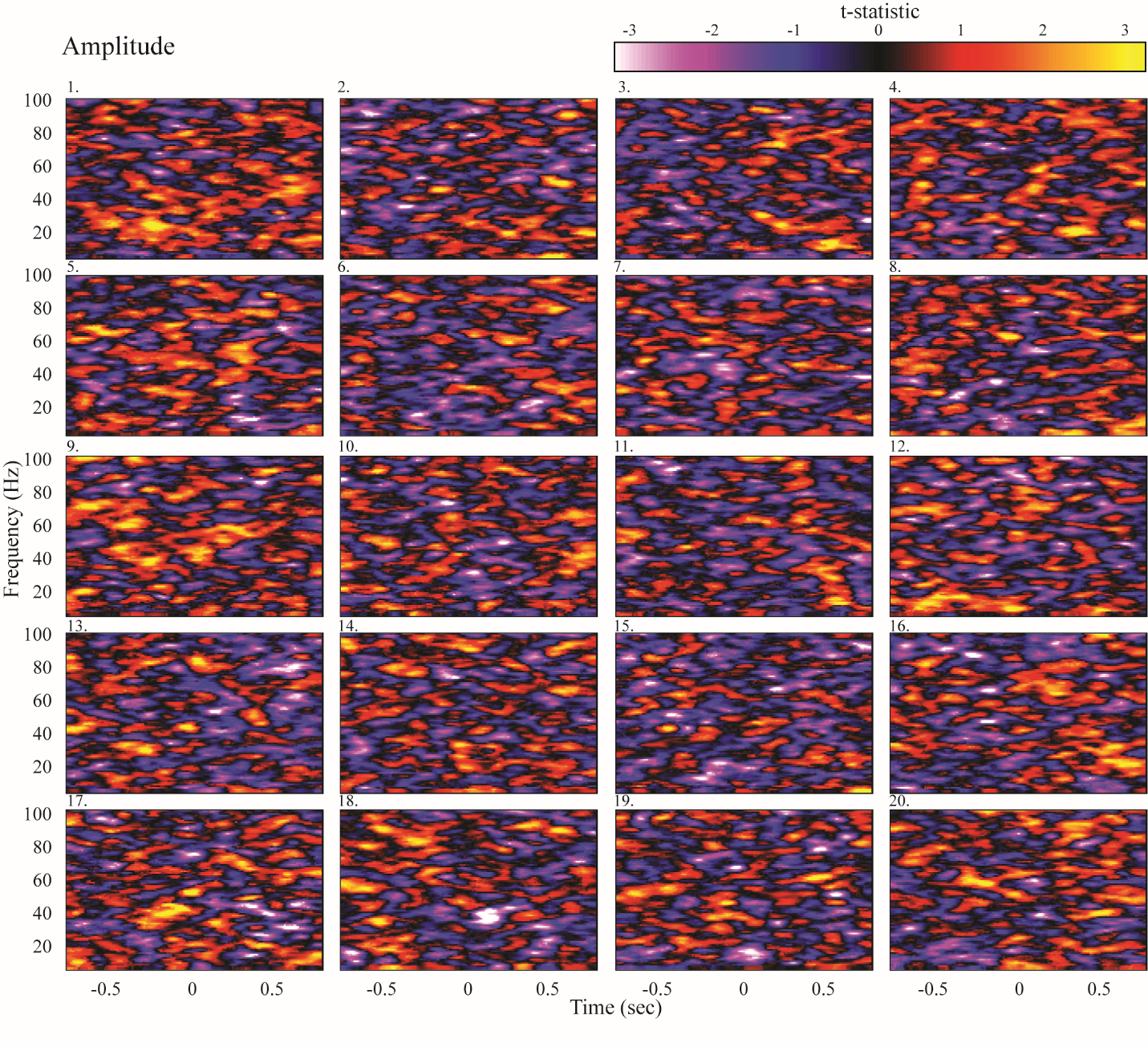


**Figure SI7.** Individual participants’ time × frequency representation of amplitude difference between successful and unsuccessful burst counting performance.


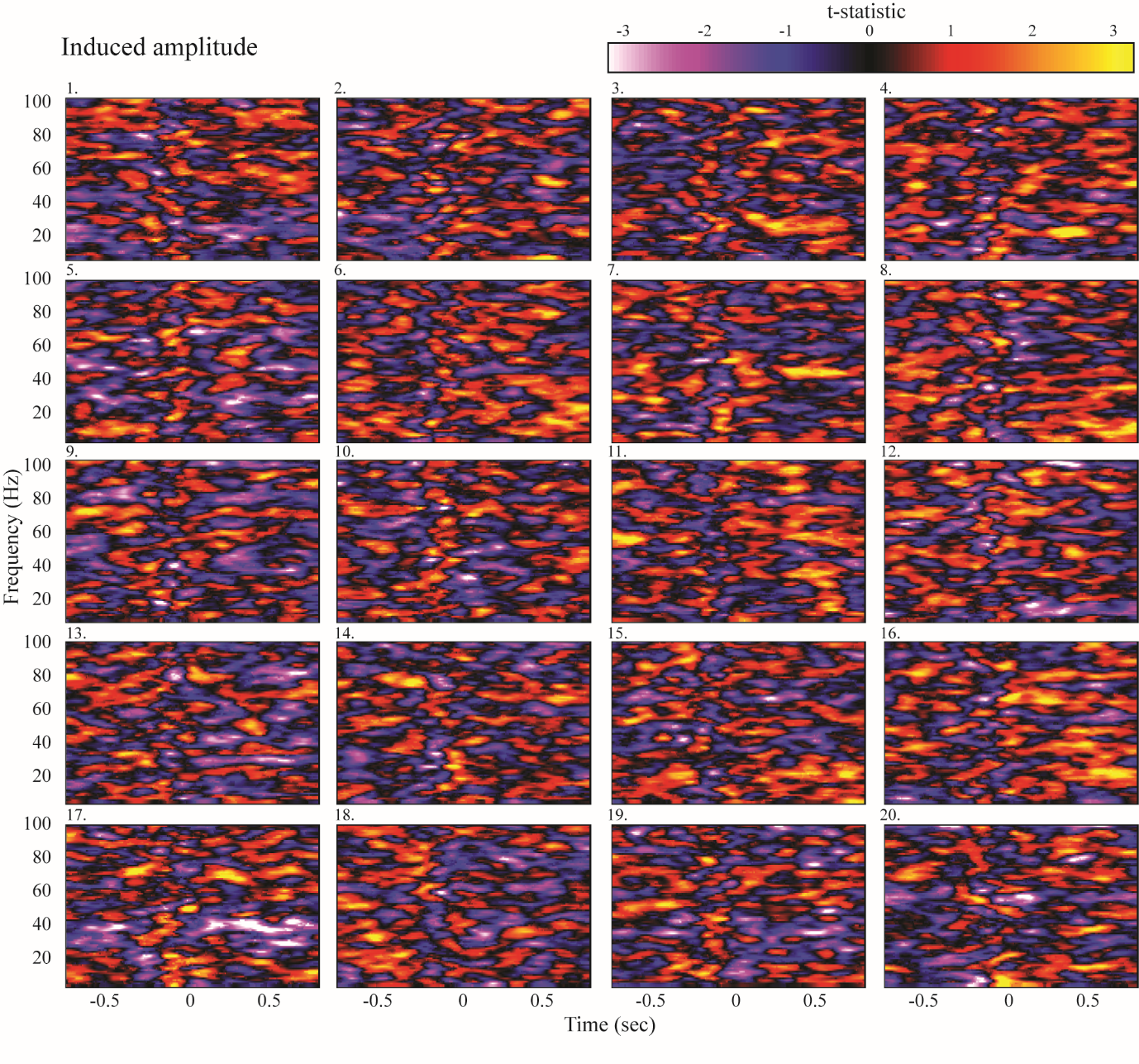
**Figure SI8.** Individual participants’ time × frequency representation of induced (baselined) amplitude difference between successful and unsuccessful burst counting performance.


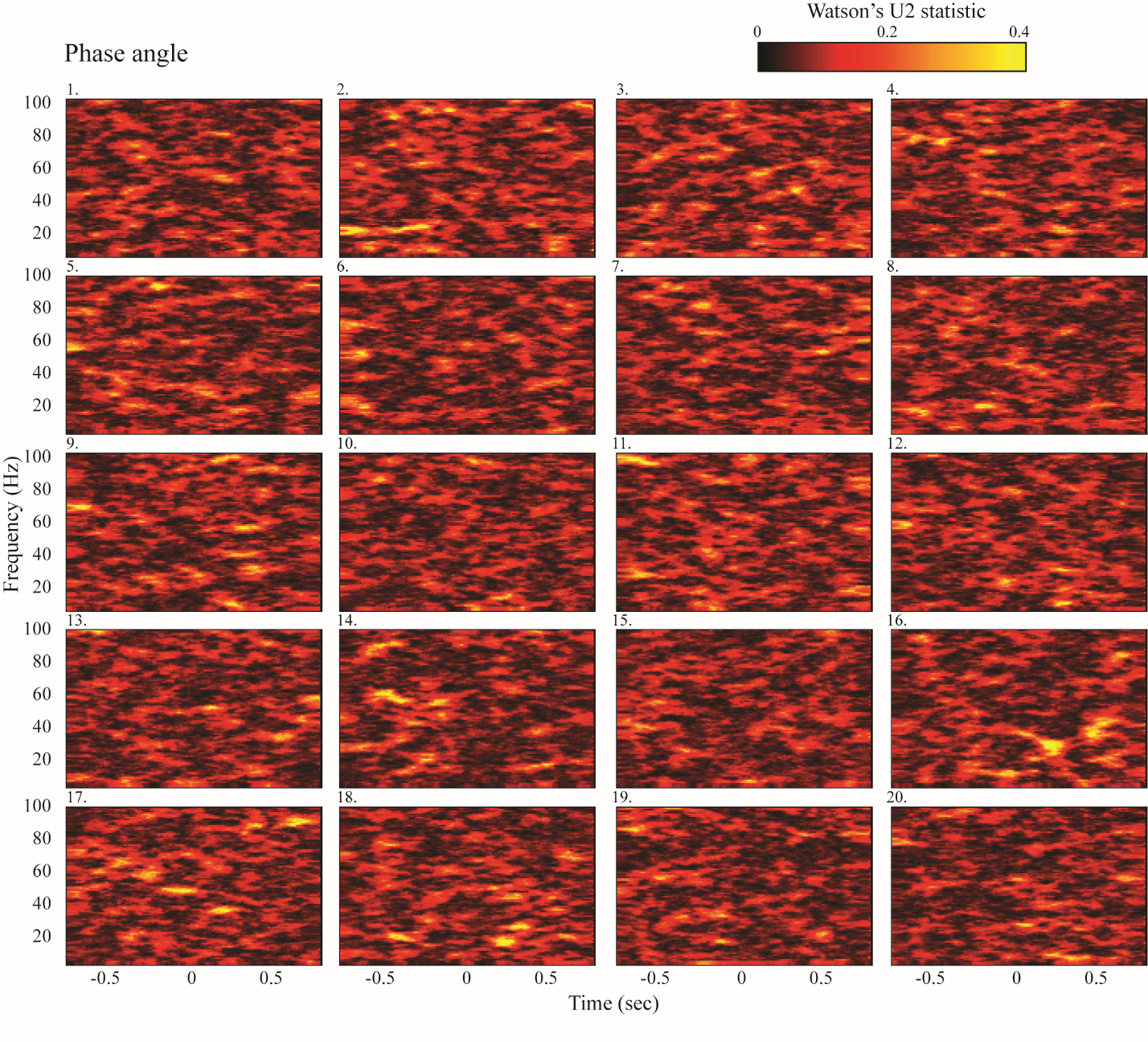


**Figure SI9.** Individual participants’ time × frequency representation of phase angle difference between successful and unsuccessful burst counting performance.


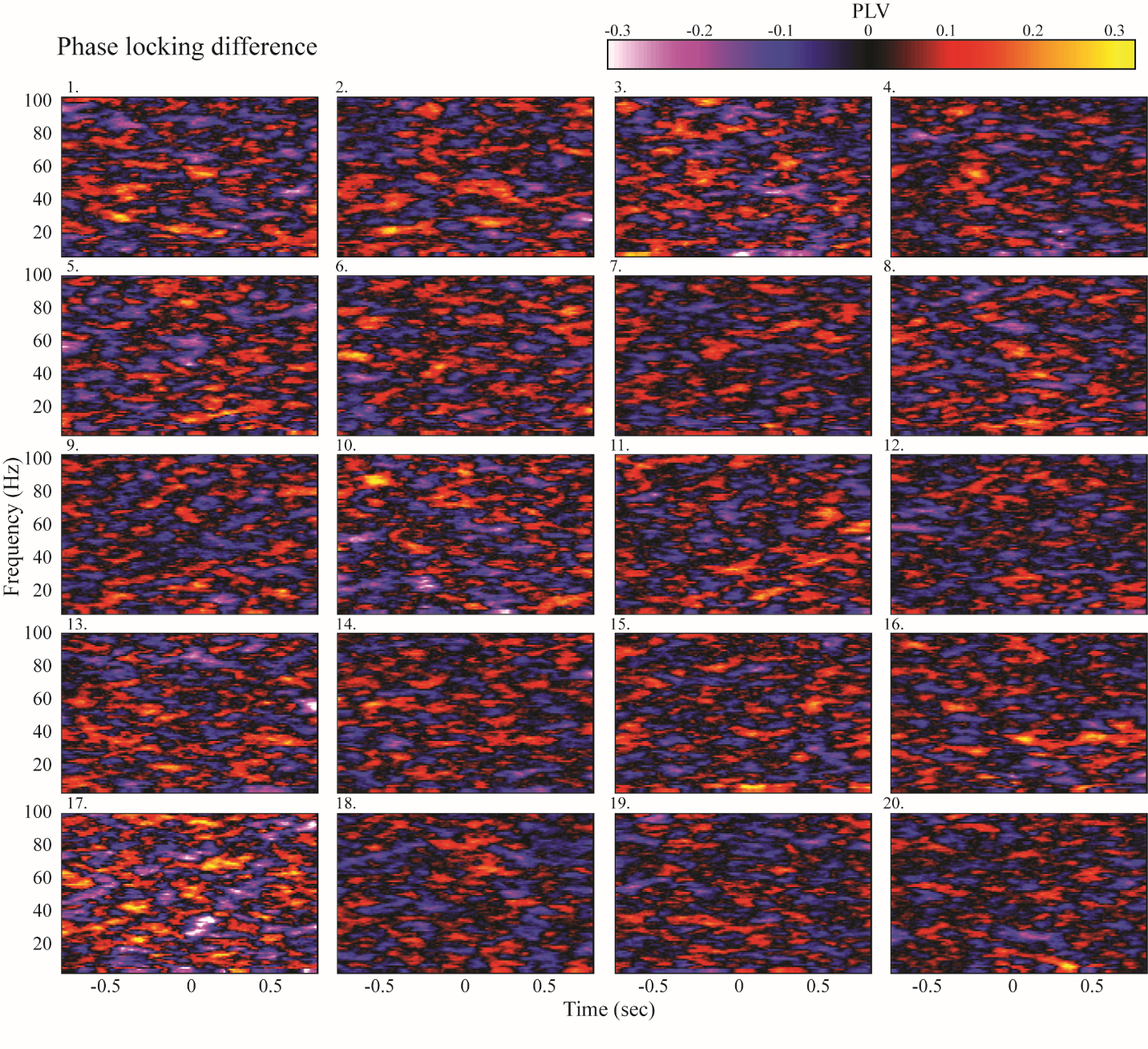


**Figure SI10.** Individual participants’ time × frequency representation of phase-locking value (PLV) difference between successful and unsuccessful burst counting performance.


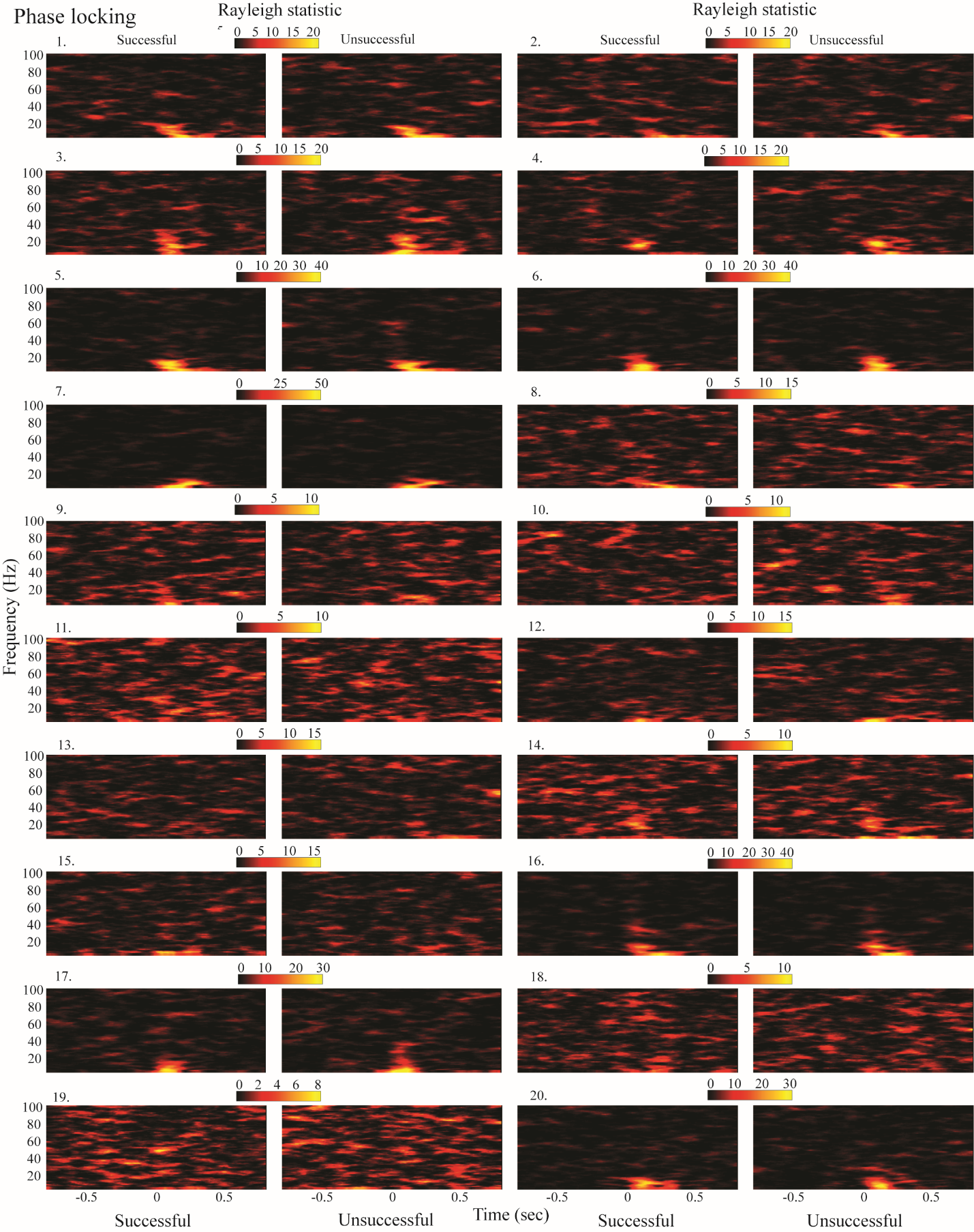


**Figure SI11.** Individual participants’ time × frequency representation of phase locking in successful and unsuccessful burst counting conditions quantified via the Rayleigh statistic.

**

Table SI3.** Summary of mass clusters applied at participant level. P values are the probabilities of there being a connected time × frequency region where significance criteria (p<0.05) is surpassed and has greater magnitude by chance for individual participants’ time × frequency spectra, applied to measures of amplitude difference, induced amplitude difference, phase angle. The range of the cluster (frequency and temporal range) are also provided.

Following the collection of the data the analyses revealed a series of γ band differences between successful and unsuccessful burst counting, a potential concern being that these could pertain to muscle artefacts. Although obvious muscle artefacts had been removed from the data a further set of exploratory analyses were undertaken to assess the spatial distribution of the differences (Muthukumaraswamy, 2013). This applied the SAM technique (Vrba & Robinson, 2001) described in the Materials and Methods, in the oscillatory domain where the parameters corresponded to the γ amplitude differences established using the pre-registered methods. The pre-stimulus γ desynchronisation covered a period from -356 to -18ms between 66 and 73Hz. The SAM analysis applied to the induced difference was baselined (-350 to 0ms), again used a 66-73Hz frequency range and an active period from 7 to 678ms relative to stimulus onset. The topographic distribution of the group level oscillatory amplitude differences between the successful and unsuccessful counted trials are depicted in Figure SI12. The peak location of the unbaselined pre-stimulus desynchronization was found within the left superior temporal gyrus (Talairach coordinates: -47.2,17.1,-25.0). The largest peak of the post stimulus difference resolved to the left lentiform nucleus (Talairach coordinates: -21.1,-5.0,-7.0) just medial to the left superior temporal gyrus, where the second peak was found (Talairach coordinates: 45.2,13.1,-25.0). As the superior temporal gyrus contains the primary auditory cortex, this topography was consistent with a cortical, as opposed to muscular, origin of the observed differences. However, the peak location of the modelled dipoles resolved to a more anterior location than one might expect of pure auditory activity (Rademacher *et al.*, 2001). The structural-functional specificity of auditory regions has been shown to exhibit a high degree of variability (Penhune *et al.*, 1996; Visser *et al.*, 2009) and the anterior location could relate to the cognitive or semantic counting comprehension aspect of the task, activating secondary auditory areas (Patterson *et al.*, 2007; Visser *et al.*, 2009). The proximity of the peaks to the eye does, however, still raise the possibility of an eye movement related artefact. However, the dipole appeared to be strongly left lateralized (see figure SI12). This, while consistent with a cognitive processing interpretation (Tervaniemi & Hugdahl, 2003), it is contrary to what would be expected of an eye movement artefact, which should present bilaterally. Muscle artefacts may also be expected to extend beyond the range of the limited (<75Hz) γ band differences observed here (see figure 5)(Muthukumaraswamy, 2013).


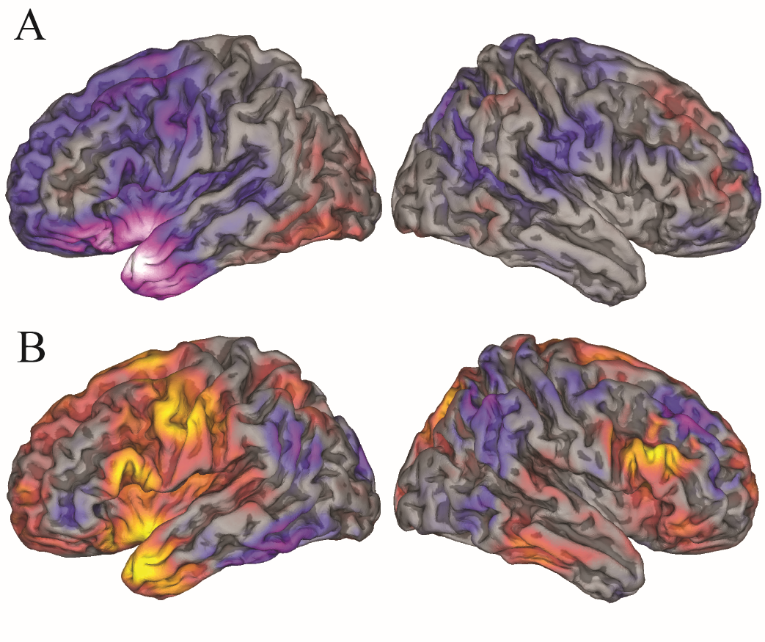


**Figure SI12.** Illustrations of spatial distribution of γ difference between successful and unsuccessful burst counting. Topographic representations are of SAM beamformer (Vrba & Robinson, 2001) solutions given parameters indicated by pre-registered analysis, for (**A**) the unbaselined difference amplitude between performance conditions between 66-73Hz over a period from -356 to 18ms seconds relative to stimuli onset and (**B**) baselined (-350 to 0ms) difference between 66-73Hz over the post stimulus period 7 to 678ms. These are group means overlaid upon semi-inflated standardized brains where blue to white colors indicate relative desynchronization in amplitude and red to yellow colors indicate relative increase in synchrony (see mri3Dx (Singh KD)).

As clarification with respect to the interpretation of the γ band observations: The three measures which expressed sequential events in the γ range suggest such oscillations may constitute, contribute or signify a particular state which is conducive to the individuation of content and task performance, as described in the main text. Previously it has been suggested that certain phases of oscillatory cycles, relative to the onset of stimuli, might allow information to be parsed (Varela *et al.*, 1981; Lakatos *et al.*, 2008; Busch *et al.*, 2009). However, these previous demonstrations have tended to concern oscillatory activity around the α range, where a relatively low number of oscillatory cycles might encompass the time × frequency region expressing a difference in capacity to individuate content. Here, by contrast, the relatively fast γ band cycles mean multiple oscillatory cycles are completed, covering many phases, over the region where the phase contrast expressed differences between the successful and unsuccessful conditions. While this does not rule out the possibility that a certain phase, at a certain time, may be conducive to content individuation, neither does it directly suggest it. Rather the suggestion from the current data is that there is a state difference, involving phase angle, between the conditions, that is conducive to individuation of bursts, but precisely what the conducive phases are is not clear from the present data. The additional possibility of the γ progression observed being coupled to particular phases in low frequencies (Lakatos *et al.*, 2008; McGinn & Valiante, 2014) is an area for further investigation. Indeed these γ events could be interpreted as being of approximately θ duration, potentially reflecting cross frequency coupling (Lisman & Jensen, 2017).

There are three additional noteworthy points with respect to the progression of the γ band events. First, the induced post-stimulus synchronization when pre-stimulus baseline is subtracted may, at least in part, be due to the subtraction of the relative desynchronization, apparent in the raw amplitude contrast, prior to the onset of the stimuli. Second, although no differences in the phase consistency contrast were apparent, when corrections for multiple comparisons were applied, there did appear to be a possible spike fluctuation in phase constancy in the successful condition at around 70Hz and at approximately 300ms prior to stimulus onset (see figure 5d). Normally such a fluctuation would be not merit note. However, because it appears at the same frequency to that of other demonstrated effects and, intriguingly, at the boundary between the phase and amplitude difference, its potential occurrence highlights it as a possible target for future research. Finally, within recent theories of predictive coding, a link has been made between γ band responses, as prediction error, to feed-forward/bottom-up processing, i.e. in responses to stimuli (Bastos *et al.*, 2012, 2015). However, as the γ progression observed here starts prior to the onset of the stimuli, there would appear to be an inconsistency. Although there are ameliorating explanations, the basic incongruity is noteworthy in a context of predictive coding where testable unique predictions are scarce (Bowers & Davis, 2012).

**S4. Post task questionnaires**

Participants were asked to complete a questionnaire after the MEG data collection. These were intended to probe individual differences in performance strategy, which could then potentially have been related to differences in the MEG data. The analyses related responses to the MEG data through a series of correlations. MEG variables were the primary measures of the three main sections where differences in the MEG data were apparent. This meant that these analyses were developed after the collection of data. Therefore, although the use of the questionnaires was pre-registered, all related analyses were developed post-hoc, and as such, are to be considered as exploratory.

The measures drawn from the main three analyses sections are as follows: The first main analyses probed the match between the behavioral distribution and the oscillatory amplitude distribution. The estimates of the extent to which individual participants displayed a correspondence between the behavioral and oscillatory distributions are summarized by their individual r coefficients and gradient (m) values. The offset between the behavioral and oscillatory distributions is summarized by the intercept (c). Correlations were also applied between the questionnaire responses and the β coefficients from the 1/frequency^β^ fits derived for behavioral or oscillatory measures separately, as well as the discrepancy between the behavioral or oscillatory distributions summarized in a β difference score. The second main analysis probed the entrainment effect, which can be summarized for each participant as a single measure of the difference in amplitude between where data is drawn from the matched frequency and the down-sampled randomized alternative set of trials. The final set of main analyses contrasted successfully individuated trials against unsuccessful trials and showed appreciable differences at the group level in three domains: raw amplitude, induced amplitude and phase angle. As the phase data is circular the correlation was restricted to amplitude differences. This used the mean amplitude difference between the successful and unsuccessful conditions across the time and frequency regions highlighted by the mass cluster-based correction (depicted in figure 5).

Prior to analyzing the relationships between the questionnaire and MEG data the following hypotheses (HQ) were drawn, for each of the questions posed to the participants. The questions (Q) as presented to the participants are given below. These are presented in italics together with the corresponding hypotheses. Coding of responses has also been provided. Numbering of questions, hypothesis and the coding have been added here for clarification but were not presented to participants. Correlations between questionnaire response and measures described above used Pearson correlation for continuous responses and Spearman's for categorical responses, the resultant r values were then used to apply a Bayesian correlation test (Wetzels & Wagenmakers, 2012).

Q1. 1A. *Did you find yourself counting the number of bursts in your head?*

*Yes [ ] No [ ]* (yes=1, no=0)

1B. *If so please make a mark on the line below to estimate how often that you adopted this strategy?*

*0%----------------------------------------50%--------------------------------------100%*

*None of the time Half of the time All of the time*

(as proportion 0-1)

HQ1. The task was designed to rely upon such a strategy of counting to individuate bursts. Therefore participants who "counted the bursts in their head" (response 1 question 1A and higher responses to question 1B) may have expressed larger effects of the stimuli in terms of both the entrainment effect and differentiation between successful and unsuccessful trials. Furthermore, as the proposal is that task performance realize upon such a strategy and this may have been facilitated by brain oscillations, it was predicted that the coupling (r and m values, of individual subjects correlations of the primary ANCOVA) may be higher in such participants.

Q2. 2A. *Did you count as the bursts were presented or look back / reflect upon the stimuli afterwards?*

*Count as they were presented [ ] Reflect afterwards [ ]*

(reflected back=1, as presented=2, both=3)

2B. *If you reflected upon the trains afterwards, how often would you estimate that you adopted this strategy?*

*0%-----------------------------------------50%--------------------------------------100%*

*None of the time Half of the time All of the time*

(as proportion 0-1)

HQ2. Participants who counted the bursts as they went along, as opposed to those who reflected back after, (response 1 for question 2A and lower responses to question 2B) might have expressed larger entrainment effects and a greater differentiation between successful and unsuccessful trials as both analyses were orientated towards differences around the period of stimuli presentation.

Q3. 3A. *Did you ever find yourself estimating the number of bursts based on the duration of the train rather than count the bursts individually?*

*Yes [ ] No [ ]* (yes=1, no=0)

3B. *If so, how often would you estimate that you adopted this strategy?*

*0%-----------------------------------------50%---------------------------------------100%*

*None of the time Half of the time All of the time*

(as proportion 0-1)

3C. *Also with respect to when you adopted this strategy, was it for;*

*all trials regardless of rate [ ], just fast trials [ ], just slow trials [ ].*

(All=1, fast=2, slow=3)

HQ3. Participants who based their responses on the duration of the burst trains rather than individuated burst (question 3A response 1 and higher responses to question 3B) might be expected to show weaker entrainment effects and differences between successful and unsuccessful trials, again owing to the temporal structure of the analyses; being based around the period surrounding burst onset. Perhaps more importantly, participants who used the train duration might be expected to have expressed a reduced coupling between the oscillatory and behavioral response distributions assayed in the first analyses. The reason being that estimation of duration may be facilitated at higher frequencies, where duration is shorter (Eisler, 1976). Adoption of this approach may have resulted in reduced correlation (lower r values) and potentially negative gradients (m values) for the fits between behavioral and oscillatory data. This might practically be the case if participants were to perform the task based on duration of trains when they are presented at lower, or across, frequencies of presentation (responses 1 and 3 to question 3C).

Q4. 4. *Please make a mark on the line below to indicate what you did when you did not know how many bursts there were. Did you*

*|-------------------------------------------------|----------------------------------------------|*

*Always give 50/50 Just press*

*your best guess? mix any button?*  (always best guess 0-1 any button)

HQ4. It is possible that participants who gave "their best guess" best guess when they were unsure as to the number of bursts, as opposed to "just pressing any button" (lower responses to question 4), may have utilized some form of residual or unconscious capacity e.g. (Sanders *et al.*, 1974). This might have increased their capacity resulting in lower behavioral β coefficients when exponential models were fitted. If, however, the oscillations perform a role in specifically conscious processing, then we might expect the discrepancy between the oscillatory and behavioral data (c intercept of the ANCOVA analysis) to be greater in these participants who may utilize unconscious capacity.

Q5. 5. *Please describe any other strategy you used, especially if the above questions do not provided an adequate description.*

*Please give any other comments you wish to make.*

No specific hypotheses or analyses were targeted at the final question.

Table SI4 describes the responses collected using the questionnaires, which can be summarized as follows: All participants responded that they "counted the numbers of bursts in their heads" (Q1A) and did so on the majority of trials (Q1B: mean proportion (±standard deviation (SD)): 0.81±0.15). A greater number of participants reported that they counted the stimuli as they were presented (12/20) compared to referring back after presentation (3/20), although 5 participants reported using both strategies (Q2A). When asked about how often they adopted the strategy of reflecting back afterward, the responses were highly variable (Q2B: 0.43(±0.20SD)). Use of the duration of the burst trains as a strategy was reportedly adopted by the majority of participants (Q3A: 15/20), but only on a relatively low proportion of trials (Q3B: 0.24(±0.22SD)) and participants reported almost exclusively doing so when stimuli were presented at the higher frequencies (Q3C: 15/16).

Table SI5 summarizes the correlations applied between the experimental measures and responses participants gave to the questionnaires. Two correlations passed the frequentist criteria level of p<0.05 without correction for multiple comparisons. One of these was a potential relationship which involved a negative correlation between the magnitude of the induced γ difference between successful and unsuccessful trials and the extent to which participants adopted the strategy of estimating the number of bursts based on the duration of burst trains (question 3B). However, this would not survive correction for multiple comparisons and did not demonstrate substantial evidence in favor of an association via the Bayesian method. Therefore no conclusions should be drawn, although it may highlight a potential area for future investigation. The other apparent correlation would not survive Bonferroni correction for multiple comparisons either, but the corresponding BF (8.18) did indicate substantial evidence in favor of an association was between the extent to which participants 'counted the number of bursts in their heads' (1B) and the gradient (or association) between the behavioral and oscillatory primary measures. This relationship was predicted and potentially suggests that participants who adopted the 'counting burst' strategy may have expressed a more pronounced correspondence between behavioral and oscillatory measures, which in turn is consistent with the overall interpretation of the experiment where discrete representation is supported by oscillations. However, the analyses of this section are all exploratory and as such should be treated with caution. Furthermore, it is apparent that the questionnaires may have failed to capture important differences of interest i.e. the phenomenology of the task. Therefore a potential avenue for further investigation with respect to tasks such as this, would be a thorough investigation of the structure of the experience of the task, linking fine grain differences in experience to MEG measures (Varela, 1996; Petitmengin & Lachaux, 2013).





**Table SI4.** Summary of participants’ responses to questionnaires. 'Nan' indicates no response was entered.





**Table SI5.** Summary of correlations applied between experiment measures and questionnaire responses. r,m and c are the results of the correlations between the behavioral and oscillatory distributions described in the first analysis. β refers to the β coefficients applied in exploration of the oscillatory (Ocil) and behavioral (Bhv) distributions and 'Diff' refers to the difference between these β values. Entrain refers to the quantification of the entrainment of oscillatory responses by the stimulus, investigated in the second main analysis and the final set of measures represent the differences in amplitude between the successful and unsuccessful trials in the induced (Ind) response to stimuli and without a trial level baseline (Amp). Questions 1B-4 are described above. Correlation r,p and BF are provided for each comparison. Highlighted are correlations where there is evidence for some form of relationship.

**S5. Exclusion criteria**

- Participants were to be excluded whose data indicated excessive movement during acquisition. This may be detected by a greater than 10mm difference in head position between acquisition blocks or more than 10% of trials being excluded on the basis of muscle/movement artefacts within any given block, as detected during initial data inspection.

- Participants who reported excessive fatigue or discomfort during acquisition were to be excluded, as would participants who fell asleep during acquisition.

- Behavioral data that indicated participants did not perform the task as instructed (e.g. fingers allocated to the wrong response buttons) would have led to the exclusion of the corresponding data.

- Other unanticipated technical failures may have led to the exclusion of data.

- Participants were free to withdraw their participation and data at any point of the investigation.

**S6. Participant instructions**

The following instructions were given to participants in written form before starting the experiment.

“You will be presented with bursts of noise, in trains of between 4 and 7 bursts. These burst trains will be at different rates. Your task is to count the number of bursts.

The response box has 4 buttons on its top which correspond to responses 4, 5, 6 and 7 going from left to right. You can take your time to respond and the next train won't start until shortly after you have made your response to the previous train of bursts. There are to be 4 blocks of burst trains each lasting approximately 8 minutes.

When doing this task please close your eyes, try to stay awake, try not to move and count the number of bursts.”

**S7. Bibliography**

Bastos, A.M., Usrey, W.M., Adams, R.A., Mangun, G.R., Fries, P., & Friston, K.J. (2012) Canonical microcircuits for predictive coding. *Neuron*, **76**, 695–711.

Bastos, A.M., Vezoli, J., Bosman, C.A., Schoffelen, J.-M., Oostenveld, R., Dowdall, J.R., De Weerd, P., Kennedy, H., & Fries, P. (2015) Visual Areas Exert Feedforward and Feedback Influences through Distinct Frequency Channels. *Neuron*, **85**, 390–401.

Benjamini, Y. & Hochberg, Y. (1995) Controlling the False Discovery Rate: A Practical and Powerful Approach to Multiple Testing. *J. R. Stat. Soc. Ser. B*, **57**, 289–300.

Benjamini, Y. & Yekutieli, D. (2001) The control of the false discovery rate in multiple testing under dependency 1165–1188.

Bland, J.M. & Altman, D.G. (1995a) Calculating correlation coefficients with repeated observations: Part 1--Correlation within subjects. *BMJ*, **310**, 446.

Bland, J.M. & Altman, D.G. (1995b) Calculating correlation coefficients with repeated observations: Part 2--Correlation between subjects. *BMJ*, **310**, 633.

Bowers, J.S. & Davis, C.J. (2012) Bayesian just-so stories in psychology and neuroscience. *Psychol. Bull.*, **138**, 389–414.

Busch, N.A., Dubois, J., & VanRullen, R. (2009) The phase of ongoing EEG oscillations predicts visual perception. *J Neurosci*, **29**, 7869–7876.

Dienes, Z. (2014) Using Bayes to get the most out of non-significant results. *Front. Psychol.*, **5**, 781.

Eisler, H. (1976) Experiments on subjective duration 1868-1975: A collection of power function exponents. *Psychol. Bull.*, **83**, 1154–1171.

He, B.J. (2014) Scale-free brain activity: past, present, and future. *Trends Cogn. Sci.*, **18**, 480–487.

Lakatos, P., Karmos, G., Mehta, A.D., Ulbert, I., & Schroeder, C.E. (2008) Entrainment of neuronal oscillations as a mechanism of attentional selection. *Science (80-. ).*, **320**, 110–113.

Linkenkaer-Hansen, K., Nikouline, V. V, Palva, J.M., & Ilmoniemi, R.J. (2001) Long-range temporal correlations and scaling behavior in human brain oscillations. *J. Neurosci.*, **21**, 1370–1377.

Lisman, J.E. & Jensen, O. (2017) The Theta-Gamma Neural Code. *Neuron*, **77**, 1002–1016.

McGinn, R.J. & Valiante, T.A. (2014) Phase-amplitude coupling and interlaminar synchrony are correlated in human neocortex. *J. Neurosci.*, **34**, 15923–15930.

Muthukumaraswamy, S. (2013) High-frequency brain activity and muscle artifacts in MEG/EEG: A review and recommendations . *Front. Hum. Neurosci.* ,.

Palva, J.M., Zhigalov, A., Hirvonen, J., Korhonen, O., Linkenkaer-Hansen, K., & Palva, S. (2013) Neuronal long-range temporal correlations and avalanche dynamics are correlated with behavioral scaling laws. *Proc. Natl. Acad. Sci. U. S. A.*, **110**, 3585–3590.

Patterson, K., Nestor, P.J., & Rogers, T.T. (2007) Where do you know what you know? The representation of semantic knowledge in the human brain. *Nat Rev Neurosci*, **8**, 976–987.

Penhune, V.B., Zatorre, R.J., MacDonald, J.D., & Evans, A.C. (1996) Interhemispheric Anatomical Differences in Human Primary Auditory Cortex: Probabilistic Mapping and Volume Measurement from Magnetic Resonance Scans. *Cereb. Cortex* , **6**, 661–672.

Petitmengin, C. & Lachaux, J.-P. (2013) Microcognitive science: bridging experiential and neuronal microdynamics. *Front. Hum. Neurosci.*, **7**, 617.

Rademacher, J., Morosan, P., Schormann, T., Schleicher, A., Werner, C., Freund, H.-J., & Zilles, K. (2001) Probabilistic Mapping and Volume Measurement of Human Primary Auditory Cortex. *Neuroimage*, **13**, 669–683.

Rouder, J.N., Morey, R.D., Speckman, P.L., & Province, J.M. (2012) Default Bayes factors for ANOVA designs. *J. Math. Psychol.*, **56**, 356–374.

Rouder, J.N., Speckman, P.L., Sun, D.C., Morey, R.D., & Iverson, G. (2009) Bayesian t tests for accepting and rejecting the null hypothesis. *Psychon. Bull. Rev.*, **16**, 225–237.

Sanders, M.D., Warrington, E.K., Marshall, J., & Wieskrantz, L. (1974) “Blindsight”: Vision in a field defect. *Lancet*, **1**, 707–708.

Shrout, P.E. & Fleiss, J.L. (1979) Intraclass correlations: uses in assessing rater reliability. *Psychol. Bull.*, **86**, 420–428.

Tervaniemi, M. & Hugdahl, K. (2003) Lateralization of auditory-cortex functions. *Brain Res. Brain Res. Rev.*, **43**, 231–246.

Varela, F.J. (1996) Neurophenomenology: a methodological remedy for the hard problem. *J. Conciousness Stud.*, **3**, 20.

Varela, F.J., Toro, A., John, E.R., & Schwartz, E.L. (1981) Perceptual framing and cortical alpha rhythm. *Neuropsychologia*, **19**, 675–686.

Visser, M., Jefferies, E., & Lambon Ralph, M.A. (2009) Semantic Processing in the Anterior Temporal Lobes: A Meta-analysis of the Functional Neuroimaging Literature. *J. Cogn. Neurosci.*, **22**, 1083–1094.

Vrba, J. & Robinson, S.E. (2001) Signal processing in magnetoencephalography. *Methods*, **25**, 249–271.

Wetzels, R. & Wagenmakers, E.J. (2012) A default Bayesian hypothesis test for correlations and partial correlations. *Psychon Bull Rev*, **19**, 1057–1064.
