# Supplementary material for: The relationship between the temporal structure of magnetoencephalography recorded brain activity and capacity to form discrete auditory representations
Source: Eur J Neurosci. 2018 Dec 11;49(12):1564–74. doi: 10.1111/ejn.14289 (PMC6618102; doi:10.1111/ejn.14289)
Supplement: Supplementary file 2 [file EJN-49-1564-s002.docx]

The relationship between the temporal structure of magnetoencephalography recorded brain activity and capacity to form discrete auditory representations

Allen, Chris.

_____________________________________________________________________________________

| Review timeline: | Submission date: 14/05/18  Editorial Decision: Reject and Resubmit  Revision Received: 19/10/18  Editorial Decision: Minor Revision  Revision Received: 02/11/18  Accepted: 09/11/18 |  |
| --- | --- | --- |
|  |  |  |

Editor: John Foxe

Reviewer 1: Xiangbin Teng

Reviewer 2: Mick Crosse

_____________________________________________________________________________________

1st Editorial Decision	13/08/18

_____________________________________________________________________________________

The separation of auditory experience and the temporal structure of magnetoencephalography recorded brain activity 

Dear Dr. Allen, 

Your manuscript was reviewed by two external reviewers as well as by the editors-in-chief. Apologies for the long delay in getting this decision letter to you, but we had considerable difficulty initially in engaging reviewers. In any case, based on these initial reviews and our own reading of the paper, we regret to inform you that we are not able to accept your manuscript for publication in EJN in its present form. However, the research described in your manuscript is potentially of interest and therefore we invite you to resubmit a much revised version. 

The basis for this decision stems from the following major issues: The overarching framework for the paper is not sufficiently elaborated and the main questions/hypotheses are not properly justified. It is felt that you have not done a sufficient job of framing the work in terms of the background literature. The paper also needs to include considerably more methodological detail – it is not sufficient to rely on the pre-registered text – the paper needs to stand alone and include all necessary details to allow for reproducibility. It has also been questioned whether you have the necessary density of data to make the measures you intend. Figure 3 clearly needs to be re-plotted and there are anomalies in the data (see Figure 5) that will need some explanation.  Reviewer #1 takes issue with the data normalization procedures and asks that the original data also be illustrated. Reviewer #2 raises the possibility that there is another plausible strategy that participants may have taken to solve the task – retrospective counting. How would this impact your results and can it be ruled out/in with additional analyses. The novelty of your entrainment results has been questioned and the fact that this has not been related to behavior needs to be addressed. 

We also find the title of the paper to be rather uninformative and obtuse. Can you please re-title the work to more directly reflect what your experiments actually tested. 

Obviously, a resubmission of your manuscript could only be considered on the basis of extensive revisions, requiring the description of new evidence and additional analyses of your data. If you do decide to take on the challenge of a re-submission, please consult the Author's Checklist provided at the bottom on this email, notably with regard to characterization of reagents and reporting of statistical analyzes in the Results section and Figure legends. 

When revising the manuscript, please embolden or underline major changes to the text so they are easily identifiable and DO NOT leave 'track change' formatting marks in your paper. If the changes made are extensive, please also provide an unmarked version. 

Please provide a detailed response to the reviewers' comments, describing the changes you have made in the text. Please upload this response letter as a separate Word (.doc) file using the file designation "Authors' Response to Reviewers" when uploading your manuscript files. 

When finalized, please upload your complete revised manuscript onto the website as a Word (.doc or .docx), or .rtf file.  Please also ensure that a complete set of tables and figures is included as separate files, even if these have not changed from the originals.  At this stage it is necessary to provide high resolution figures.  Please see important instructions below. 

To re-submit your manuscript, please go to your Author Centre and check in 'Manuscripts with decisions' under the previous manuscript number for a 'Create a resubmission' link.  Also, please note that only the Author who submitted the original version of the manuscript should submit a revised version. 

If you feel that your paper could benefit from English language polishing, you may wish to consider having your paper professionally edited for English language by a service such as Wiley’s at http://wileyeditingservices.com. Please note that while this service will greatly improve the readability of your paper, it does not guarantee acceptance of your paper by the journal. 

Thank you for submitting your work to EJN. 

Kind regards, 

John Foxe & Paul Bolam 
co-Editors in Chief, EJN 


Reviews: 

Reviewer: 1 

Comments to the Author 
The author conducted a MEG study with noise bursts presented at different rates to investigate how auditory experience is separated or discretized.  The experiment appears to be probably conducted and data analysed adequately, but it is hard to see what the author would like to test from the manuscript. The manuscript is loosely written without adequate background information. And many procedures are not well motivated. It is unclear what the study tells readers.   


Major comments 
1. The abstract and introduction of this manuscript provide little information on what questions the author aimed to answer or test. Maybe I am missing something here. I see that in the pre-registered form, the introduction part is relatively more comprehensive. Is this ok to omit a large part of information here? However, even by reading both the pre-registered form and the manuscript, one may still feel that the introduction lacks a considerable amount of information. For example, the auditory stimuli were used, but no relevant studies were cited or referred to. I get the sense that the author might want to test sensory discretization in the auditory domain, but it would be very surprising that there are no relevant studies needed to be discussed or compared with.  This probably renders the conclusion of this study inconclusive. It is hard to see what the main message of this study is. 
  
2. Maybe because this study was pre-registered, the method section is not detailed at all. From the manuscript along, it is hard to see how the noise bursts were generated. And what is the sampling rate of the stimuli? any filters are used? and what kind of noise, white or pink? This made people go back and forth between the manuscript and the pre-registered form to find out what were done here. This isn’t helping readers. This is also the case in the following texts. For example, no information was provided for what kinds of bandpass filters were used to extract phase and power. By reading the method section, one may have a feeling that it is not clear what was done here. More details would help a lot. 

3. Figure 3. It can be plotted as a figure of double y axes, with one for amplitude and one for behavioural results. It is somehow not common to normalize the behavioural data this way. I would like to see the raw behavioural data. Can subjects really count the bursts at the rate of 8 Hz? If yes, what is the percentage correct? A lot of information was hidden behind this normalization procedure. 

4. Do the author have a good explanation on why some effects in figure 5 are shown even before the stimuli are presented. This can puzzle readers and makes people wonder whether there is something problematic with analysis or statistic tests.   

5. If I understand it correctly, most of stimuli were only presented 8 times (‘at least 8 times’), I am not sure it is enough. Can the author show an onset response to check whether such a small amount of trials give reliable signal to noise ratio? 

6. If here the counting task is used to probe sensory discretization and is correlated with meg data, readers may wonder whether the author thought this counting task can be used this way. Any explanations on that? 


Minor comments 
1 If the author could include line numbers, it would make review process easier. 
2. Abstract, line 1, ‘activity’ and  ‘representation’ appear to have very general meanings here.   
3. Abstract, ‘a broadband low-level function of cortical oscillations’. I cannot really understand this phrase. 
4. abstract, last sentence, ‘impacts’ or ‘impact’? 
5. what version of psychophysics toolbox. Any references here? 
6 why did the author use a symbol to indicate ‘inter-onset intervals between bursts’. Is this more straightforward? 
7  It can be very helpful to have an illustration of stimuli like in figure 2. Or, figure 1 and 2 can be put together. This would make readers easily understand what the stimuli are. 


Reviewer: 2 

Comments to the Author 
Summary 
The present study investigated the relationship between the alignment of oscillatory brain activity and the capacity to form discrete auditory representations. To examine this question, an MEG protocol was developed and three sets of analyses were pre-registered and carried out. The first analysis demonstrated that the capacity to form auditory representations correlated with auditory cortical activity. The second analysis showed that auditory cortical oscillations entrained to stimuli over a broad range of frequencies. The third found that activity in the gamma band predicted successful representation. These findings were interpreted as evidence that a low-level function of cortical oscillations is to set conditions for representational neuronal assemblies in a frequency non-specific manner, and that this likely impacts the temporal dynamics of experience. 
The research questions investigated here are indeed of great importance to our understanding of the functional significance of cortical oscillations broadly speaking, and the neural mechanisms underlying auditory representation and the flow of experience in general. The study focused on the three pre-registered analyses, incorporated certain adjustments as necessary (all of which were well justified) and included additional exploratory analyses as supporting information. While this approach to science is exemplary, I have some major concerns with the approach to some of the analyses, their novelty and interpretation. I have outlined my concerns below and would appreciate if the authors could address these, and/or include additional analyses as required. Clarification is also needed in much of the methods section as outlined below. 
Major comments 
The correlation analysis, from my interpretation, was conducted within subjects, measuring the relationship between accurate counting performance and prevalence of oscillatory activity across frequency bands. My issue with this analysis is the following: given that MEG/EEG typically presents with the spectral profile observed here (i.e., most of the power below ~10 Hz – although I acknowledge that the authors reference instances where it did not in the discussion) and given that it gets more difficult to count periodic stimuli at faster rates, is it any wonder that each participants MEG and behavioral functions were correlated? Would it not have been better to examine the correlation between behavior and MEG separately at each frequency band and across participants? Then you could be sure that oscillatory activity covaried with performance independent of the inherent spectral profile of MEG and behavior. 
Having listened to the stimuli online, it appears to me that the presentation rate is often too fast to count the stimuli in real time, and that is easier to replay the stimulus in your head after you’ve heard it and then count the number of bursts. How may memory and/or musical or rhythmic ability have affected the behavioral results? Was there a particular counting strategy instructed to the participants or reported by the participants? Moreover, if counting was done after listening to the stimuli, would it not have been important to focus the analysis on a post-stimulus window as opposed to the chosen epoch which coincided with the stimulus duration? 
The second hypothesis states that cortical oscillations should become entrained to the frequency of the stimuli and in doing so provide opportunities for representation. However, entrainment of oscillatory cortical activity to periodic acoustic stimuli is a well-established phenomenon and thus does not provide any novel information. Furthermore, the authors do not link this directly to behavior, rather, they hypothesize that in evoking such entrainment that it should necessarily enhance auditory representation. While I do not dispute this, it would be nice to see a correlational analysis between entrainment and behavior to further bolster this theory and improve the overall impact of this analysis. 
Minor comments 
Title 
Why use the term “separation” when the paper sought to establish a functional relationship between cortical oscillations and auditory experience? 
Intro 
Page 2: “have met with” should be “have been met with” 
Page 3: What does “designed to tack oscillations” mean? 
Methods 
Page 4: How did the participants report the number of bursts counted each time. 
Page 4: Can the authors be more explicit about what they mean by “merging if they failed”. 
Page 5: Why was an epoch size of -800 to 800 ms chosen if this wasn’t big enough to capture the length of say a 6 Hz stimulus with 7 bursts, e.g., Fig. 2? The active period was supposed to range from “the onset of the first burst until 200 ms after the end of the final burst”. Could this have biased the analysis for stimuli that fit inside the chosen epoch? 
Page 5: Why were different active periods used for the correlation and entrainment analyses? Can the authors be clear as to why different techniques were used to measure oscillatory prevalence and oscillatory amplitude and how they differ? Or were they both the mean amplitude of the Hilbert transform? Supporting information seems to indicates so but it is not clear in the main text. 
Page 7: Should the second “BF>3” be “BF<-3”? 
Page 8: Typo – comma after Gross et al. (2013). 
Page 8: What do the authors mean by “point values for oscillatory amplitude”? 
Results 
Page 12: Please state explicitly the result of the relationship between subject’s individuation performance and oscillatory make-up instead of posing the question and reporting a test statistic. 
Discussion 
Page 17: typo – “...and but...” 
Supporting Information 
Page 25: typo – “...analysis programs form this experiment...” 
Page 25: typo – “These uses the same...” 

------------------------ 

____________________________________________________________________________________

Authors’ Response	19/10/18

_____________________________________________________________________________________

Dear Editors, 
I would like to thank you and the reviewers for providing such an insightful and useful set of reviews. I have made extensive revisions to the manuscript in accordance with the suggestions. These have focused upon framing the study and methodological precision independently of the pre-registration document. The title, abstract and introduction have been heavily revised. The introduction in particular has been expanded with an emphasis on providing a fuller rationale for the study and contextualising it in relation preceding literature. In my response to reviewers and the revised manuscript, I have clarified the multi-block experimental design in order to address the concerns related to data density. I have re-plotted the data as requested, presenting the data with and without the normalisation procedure applied. The perceived anomalies in the data are also addressed, as well as questions relating to alternative task strategies. In line with the reviewer’s suggestions I have also added additional analysis, below and to the manuscript, relating to the entrainment effect to behaviour. I hope you will find the amended manuscript an improvement and consider it for publication. In addition to the plain formatted main manuscript, I have provided a version of the manuscript with additions marked in bold, as requested, and uploaded it as a supporting document. Below is a point-by-point response to the reviewers’ comments. 

“Reviewer: 1 
1. The abstract and introduction of this manuscript provide little information on what questions the author aimed to answer or test. Maybe I am missing something here. I see that in the pre-registered form, the introduction part is relatively more comprehensive. Is this ok to omit a large part of information here? However, even by reading both the pre-registered form and the manuscript, one may still feel that the introduction lacks a considerable amount of information. For example, the auditory stimuli were used, but no relevant studies were cited or referred to. I get the sense that the author might want to test sensory discretization in the auditory domain, but it would be very surprising that there are no relevant studies needed to be discussed or compared with. This probably renders the conclusion of this study inconclusive. It is hard to see what the main message of this study is. “ 

I am grateful to the reviewer for highlighting this as, in responding to these suggestions by including further background information, I believe the introduction is improved. Part of the reason for minimising the discussion of background literature was to allow for the possibility of the article being published as a short report. As the manuscript is now in research report format I gladly include further background information explaining the motivation for the study and outlining more of the most relevant previous research connecting discrete representation to oscillatory brain activity. 

“2. Maybe because this study was pre-registered, the method section is not detailed at all. From the manuscript along, it is hard to see how the noise bursts were generated. And what is the sampling rate of the stimuli? any filters are used? and what kind of noise, white or pink? This made people go back and forth between the manuscript and the pre-registered form to find out what were done here. This isn’t helping readers. This is also the case in the following texts. For example, no information was provided for what kinds of bandpass filters were used to extract phase and power. By reading the method section, one may have a feeling that it is not clear what was done here. More details would help a lot.” 

All methodological details requested by the reviewer have been added to the manuscript. Stimuli details have been added to page 6. Filter information has been clarified on pages 7/8.Further additions to the methods have been made with a view to promoting comprehension and reproducibility. 

“3. Figure 3. It can be plotted as a figure of double y axes, with one for amplitude and one for behavioural results. It is somehow not common to normalize the behavioural data this way. I would like to see the raw behavioural data. Can subjects really count the bursts at the rate of 8 Hz? If yes, what is the percentage correct? A lot of information was hidden behind this normalization procedure.” 

Following this suggestion, a double y axis plot using the raw behavioural and amplitude data has been added to the manuscript (figure 3). Rather than replacing the original plot this has been included as an additional panel so that the measures to which the analyses were applied are presented. I thank the reviewer for this helpful suggestion which has resulted in a more complete exposition of the data. I can confirm that participants were able to count the bursts at 8Hz with a mean performance level of 82% (±21%SD) relative to chance performance at 25% and further information about raw performance levels has been added to the start of the results section. 

“4. Do the author have a good explanation on why some effects in figure 5 are shown even before the stimuli are presented. This can puzzle readers and makes people wonder whether there is something problematic with analysis or statistic tests.” 

Pre-stimulus differences in oscillatory state have previously been reported to be associated with successful task performance (e.g. van Dijk et al., 2008; Romei et al., 2010). With respect to the gamma band differences, as here, pre-stimulus differences have previously been linked with a state of preparedness or allocation of attention before the stimulus arrives that allows for successful task performance (Womelsdorf et al., 2006; Reinhart et al., 2011; Todorovic et al., 2011). The suggestion that such pre-stimulus gamma band fluctuations might reflect preparedness based on previous research can be found on pages 16/17. Therefore the dependence of the systems state prior to stimulus presentation to its susceptibility to representation is supported by previous research. Here the novel demonstration of a progression of gamma band oscillatory events advances understanding by suggesting that a sequence of interrelated gamma band features, expressed over a range of measures, support successful task performance. 

“5. If I understand it correctly, most of stimuli were only presented 8 times (‘at least 8 times’), I am not sure it is enough. Can the author show an onset response to check whether such a small amount of trials give reliable signal to noise ratio?” 

The manuscript has been amended to clarify the trial numbers used (page 6). “[A]t least 8 times” refers to each frequency within a single experimental block and there were four experimental blocks resulting in at least 32 presentations. Furthermore, most analyses were applied across frequencies of presentation or to the higher prevalence frequencies of presentation (128 trials per participant, per frequency). The only analyses to make use of the lower prevalence frequency conditions (32 trials per participant per frequency) was analysis 2. Here figure 4B illustrates an onset response in the form of an induced change using one of the low prevalence trail conditions. Further analyses designed to assess the impact of low trial numbers and down-sampling are described in section SI2 and pre-registered criteria designed to ensure sufficient data density are described in SI3. 

“6. If here the counting task is used to probe sensory discretization and is correlated with meg data, readers may wonder whether the author thought this counting task can be used this way. Any explanations on that?” 

The reviewer is correct to highlight this reasoning. In the genesis of this experiment the reasoning was along the following lines: If oscillations provide conditions for representation then they should place a limit upon how quickly discrete representations can form. A task which could tap into this would be one that involves rapidly forming discrete representations based on stimuli that were capable of merging. The counting task was developed because burst counting requires the capacity to form discrete representations and therefore should correlate with the MEG data. Revisions have been made to the manuscript (pages 3, 5 and 6) that I hope will make this point more clearly. 

“Minor comments 
1 If the author could include line numbers, it would make review process easier.” 

I agree with the reviewer, but the European Journal of Neuroscience guidelines states that lines should not be numbered. 

“2. Abstract, line 1, ‘activity’ and ‘representation’ appear to have very general meanings here. “ 

Alterations have been made to the first line of the abstract to clarify the terms ‘activity’ and ‘representation’, as follows: 
‘A function of oscillatory brain activity may be to align activity relative to threshold potentials and provide opportunities for representational neuronal assemblies to form.’ 

“3. Abstract, ‘a broadband low-level function of cortical oscillations’. I cannot really understand this phrase.” 

The corresponding phrase in the abstract has been clarified and now reads as follows: ‘Together, these findings indicate that a low-level function of cortical oscillations, which may apply across a range of frequency bands, is to periodically set conditions in which representational neuronal assemblies can manifest, limiting and so affecting the flow of experience.’ 

“4. abstract, last sentence, ‘impacts’ or ‘impact’?” 

‘Impact’ was the correct term, but the sentence has been altered and no longer contains the words in question. As with other points raised by the reviewer I am grateful for these helpful suggestions. 

“5. what version of psychophysics toolbox. Any references here?“ 

Version information and references have been added as suggested (page 6). 

“6 why did the author use a symbol to indicate ‘inter-onset intervals between bursts’. Is this more straightforward?” 
Lambda was used for brevity and because Lambda is commonly understood to indicate wavelength and is therefore appropriately used to denote the wavelength of the burst cycle in figure 1. The legend of figure 1 has been amended to clarify this point. 

“7 It can be very helpful to have an illustration of stimuli like in figure 2. Or, figure 1 and 2 can be put together. This would make readers easily understand what the stimuli are.” 

The legend supporting figure 1 has been amended with the aim of making the stimuli presentation more easily comprehensible. 

“Reviewer: 2 
Major comments 
The correlation analysis, from my interpretation, was conducted within subjects, measuring the relationship between accurate counting performance and prevalence of oscillatory activity across frequency bands. My issue with this analysis is the following: given that MEG/EEG typically presents with the spectral profile observed here (i.e., most of the power below ~10 Hz – although I acknowledge that the authors reference instances where it did not in the discussion) and given that it gets more difficult to count periodic stimuli at faster rates, is it any wonder that each participants MEG and behavioral functions were correlated? Would it not have been better to examine the correlation between behavior and MEG separately at each frequency band and across participants? Then you could be sure that oscillatory activity covaried with performance independent of the inherent spectral profile of MEG and behavior.” 

The reviewer is correct that given typical power spectra and that task performance becomes more difficult as frequency increases, it is unsurprising that they correlate to some extent. The manuscript has been amended to emphasise this point more fully (see 3.1 pages 12/13). However, it is also worth pointing out that the predictions also referred to the direct correspondence between behavioural capacity and oscillatory activity over a limited range of frequencies where at least one oscillatory cycle may be required to individuate content. This is independent of the concern raised, and illustrated by the overlay presented in figure 3 and the intercept of the ANCOVA approximating to zero. Furthermore, had an alternative strategy been adopted by participants (e.g. of judging the number of bursts based on train duration, rather than discretely counting them) then the task would not necessarily have been easier at lower frequencies and the correlation might not have been observed. Likewise, had performance transitioned between ceiling and floor beyond the range of innate cortical oscillatory frequencies no such correlation would have been observed. These possibilities are considered further in section SI4. 
The correlational analysis suggested by the reviewer is described in section SI1 page 6 and table SI1. However, it is difficult to draw any strong conclusions from these secondary analyses as the experiment was not powered or designed to test individual differences in this way. Furthermore, while the correlations suggested by the reviewer did not demonstrate an individual differences relationship when tested at each frequency or when collapsed across frequencies of presentation, the participant factor of the main model (which arguably utilises the most data) did indicate predictive value of participant differences (see SI1). 

“Having listened to the stimuli online, it appears to me that the presentation rate is often too fast to count the stimuli in real time, and that is easier to replay the stimulus in your head after you’ve heard it and then count the number of bursts. How may memory and/or musical or rhythmic ability have affected the behavioral results? Was there a particular counting strategy instructed to the participants or reported by the participants? Moreover, if counting was done after listening to the stimuli, would it not have been important to focus the analysis on a post-stimulus window as opposed to the chosen epoch which coincided with the stimulus duration?” 

I am grateful that the reviewer has taken the time to experience the stimuli and I find these possibilities interesting and insightful. I have been developing future experiments to test similar possibilities, looking at the experience and memory of the burst individuation process with a phenomenologically-informed interview technique (Petitmengin, 2006). I would also like to test the possibility that skilled percussionists, for example, might exhibit a different behavioural and oscillatory spectral profile in comparison to non-experts. While almost all reports of internal states in relation to stimuli to some extent depend upon memory (e.g. Block, 1995), longer inter-trial intervals together with a more nuanced report procedure may allow future experiments to specifically target the neural correlates of fine grained differences in strategies and experiences of the task. 
Although comprehensive tests of the possibility raised by the reviewers are not possible with the current data set, the questionnaire data (see SI4) does explore some strategies participants may have used in performing the task, including replaying the stimuli in memory (also see SI6). The majority of participants (12/20) reported counting the bursts as they we presented rather than referring back afterward. 3/20 reported referring back afterwards and 5/20 reported using both strategies, although as this was not an explicit option in the questionnaire the true incidence of adopting both strategies may have been higher. The reported use of one or other of these strategies did not correlate with any of the MEG-based measures. However, there was evidence that participants who reported that they “counted the number of bursts in your head” to a greater extent (question 1B), were those who demonstrate the higher gradient coefficients, quantifying stronger coupling between behavioural and oscillatory measures. Although these analyses are exploratory in nature, they do provide some response to the questions raised by the reviewer, as the relationship between oscillations and performance did appear to be underlined by “counting the bursts in your head”. 
Another aspect to these points is that the retrospective alternative strategies need not detract from or alter the interpretation presented. This is because even if performance is mediated through memory (as are all behavioural responses to stimuli, to some extent), the memory mediated response would necessarily rely upon an initial representational trace in which the stimuli were discretely represented in some way, for a successful report to be made. This is consistent with, and does not detract from, the interpretation offered. 

“The second hypothesis states that cortical oscillations should become entrained to the frequency of the stimuli and in doing so provide opportunities for representation. However, entrainment of oscillatory cortical activity to periodic acoustic stimuli is a well-established phenomenon and thus does not provide any novel information. Furthermore, the authors do not link this directly to behavior, rather, they hypothesize that in evoking such entrainment that it should necessarily enhance auditory representation. While I do not dispute this, it would be nice to see a correlational analysis between entrainment and behavior to further bolster this theory and improve the overall impact of this analysis.” 

The reviewer is correct to point out that entrainment of oscillatory activity to stimuli has previously been demonstrated. Here the original analyses demonstrated that the cortex responded to the stimuli in a frequency-specific way and that the virtual sensor and analysis approach captured this physiological response. Had such an effect not been observed the reliability of the experiment as reflecting a cortical response might have been brought into question. 
The suggestion made by the reviewer to extend this by performing a correlational analysis between entrainment and behaviour is insightful and has been implemented. I have interpreted the suggestion as a correlation between the behavioural performance measure and the difference between matched and unmatched amplitude measures from the second set of analyses (quantifying the entrainment effect), within each frequency of interest and averaged across frequencies. This tested the possibility that if entrainment was critical to performing the task of counting bursts then individual differences in behavioural performance may have correlated with the degree of entrainment. However, the results were inconclusive (using mean across frequencies r= 0.35, p=0.13, BF10=1.49*, also see table below). While there is a weak trend in the theoretically plausible direction based on the averaged data, no clear conclusions can be drawn with respect to these analyses. This is likely to be due to the paradigm being under-powered to test such an exploratory correlation. Additionally, there are several theoretical contingencies that might limit the expression of such a relationship. For example, while a degree of entrainment may support discrete representation, the third analysis suggests that successful task performance may also depend on higher frequency specific systems. Alternatively, the degree of entrainment quantified might also be mediated by other, unaccounted for, factors such as conduction properties. As these potential mediating factors are not captured by the correlational entrainment analyses, the extent to which the entrainment effect correlates with behavioural performance may be limited. I would like to thank the reviewer for this thought-provoking suggestion, the full implementation of which can now be found in supplementary section SI2. 


F (Hz) r p BF10 
3 -0.26 0.28 0.14 
4 0.08 0.74 0.37 
6 0.43 0.06 2.74 
8 0.15 0.53 0.48 
10 -0.01 0.95 0.27 
12 0.09 0.71 0.38 
14 -0.10 0.68 0.21 
16 -0.10 0.68 0.21 
18 0.18 0.45 0.55 
22 0.24 0.30 0.77 
28 -0.33 0.16 0.13 
38 0.01 0.98 0.28 
mean 0.35 0.13 1.50 

The table above summarises the exploratory correlation between the entrainment effect and behavioural performance. The entrainment effect was quantified as the difference between by matched and unmatched amplitude measures (the primary variable of analyses 2) and the performance measures was the normalised proportion correct. Presented are the correlation coefficients and corresponding p and BF10 values at each frequency (F) and using the mean across frequencies. Bayes Factors (BF10)* were derived using JASP (0.8.1, applying Pearson’s rho correlation with a positive paired prior). 

“Minor comments 
Title 
Why use the term “separation” when the paper sought to establish a functional relationship between cortical oscillations and auditory experience?” 

I now recognise the ambiguity in the title and have changed it accordingly. 

“Intro 
Page 2: “have met with” should be “have been met with”” 

The corresponding sentence has been changed accordingly. 

“Page 3: What does “designed to tack oscillations” mean?” 

This phrase has been replaced by a more detailed description of the stimuli designed in relation to the experimental goals. 

“Methods 
Page 4: How did the participants report the number of bursts counted each time.” 

Responses were collected via a Lumitouch response box. The response mapping has been made explicit on the amended version. 

“Page 4: Can the authors be more explicit about what they mean by “merging if they failed”.” 

The white noise used in the construction of the stimuli was designed to make the transition between one burst and the next minimal if discrete representation failed, leading to lower task performance. The manuscript has been amended to clarify this (see page 6). When developing the task a number of alternative stimuli were considered, but the alternatives were more problematic (e.g. ambiguous frequency structure). This is an area of ongoing development for future work. 

“Page 5: Why was an epoch size of -800 to 800 ms chosen if this wasn’t big enough to capture the length of say a 6 Hz stimulus with 7 bursts, e.g., Fig. 2? The active period was supposed to range from “the onset of the first burst until 200 ms after the end of the final burst”. Could this have biased the analysis for stimuli that fit inside the chosen epoch? 
Page 5: Why were different active periods used for the correlation and entrainment analyses? Can the authors be clear as to why different techniques were used to measure oscillatory prevalence and oscillatory amplitude and how they differ? Or were they both the mean amplitude of the Hilbert transform? Supporting information seems to indicates so but it is not clear in the main text.” 

Thank you for highlighting these ambiguities. Amendments have been added to clarify these points (pages 6-7 and 9). The reason for choosing the -800 to 800ms epoch was a balance of practical considerations which were described in the pre-registered protocol but omitted from the manuscript for brevity. These have been reinstated in the revised submission. Briefly, the -800:800ms duration meant that trials could be epoched independently while minimising carry over from preceding trials into baselines and without the introduction of long inter-trial intervals, which had the potential to affect participants performance. Additionally, for the analyses targeting differences over a range of frequencies of presentation (1 and 3), it was necessary to apply a consistent structure as varying contribution made to these analyses according to frequency of presentation had the potential to introduce biases into the data as some frequencies would contribute more data than others. Furthermore, time frequency cluster based correction could not have been applied to inconsistently epoched durations. However, for analyses which were applied to individual frequencies of presentation it was possible to adapt the active and baseline periods to the stimuli, thus utilising more of the data. This was implemented in analysis 2. In reference to analysis 2, the periods involved included an additional 200ms in order to capture the later responses to the stimuli but these were also limited to a maximum of 800ms, again to avoid inclusion of data pertaining to the preceding or subsequent trials. As equal periods were applied across the primary contrast of interest (matched vs unmatched) I do not think this could have biased the analysis. With respect to the frequency factor, of secondary interest, as lower frequencies have proportionally longer durations I think it unlikely that they biased the corresponding secondary analysis to any frequency and, while it is also conceivable that the application of the 800ms cut off could have led to frequency specific effects, no such effects were observed or discussed. The use of the Hilbert transform as applied to all measures has been clarified (page 8) and a sentence clarifying the difference between prevalence and induced amplitude related measures has been added (page 9). 

“Page 7: Should the second “BF>3” be “BF<-3”?” 

I believe the second BF>3 to be correct as an application of the default (Rouder et al 2009) Bayesian t-test method, as it is essentially a two tailed test where BF>3 would have indicated evidence in favour of a dissociation between behavioural performance or oscillatory amplitude in either direction. 

“Page 8: Typo – comma after Gross et al. (2013).” 

Thank you for pointing this out. 

“Page 8: What do the authors mean by “point values for oscillatory amplitude”?” 

Point value here refers to the averaging of the data across the active period resulting in single value estimates for the induced response. The manuscript has been amended to clarify this point (page 9). 

“Results 
Page 12: Please state explicitly the result of the relationship between subject’s individuation performance and oscillatory make-up instead of posing the question and reporting a test statistic.” 

The question structure of the corresponding analysis on Page 13 has been amended in accordance with the reviewer’s suggestion. 

“Discussion 
Page 17: typo – “...and but...” 
Supporting Information 
Page 25: typo – “...analysis programs form this experiment...” 
Page 25: typo – “These uses the same...”” 

These errors have been corrected and I thank the reviewer for highlighting them. 


References: 
Block, N. (1995) How Many Concepts of Consciousness - Authors Response. Behav. Brain Sci., 18, 272–287. 
Petitmengin, C. (2006) Describing one’s subjective experience in the second person: An interview method for the science of consciousness. Phenomenol. Cogn. Sci., 5, 229–269. 
Reinhart, R.M.G., Mathalon, D.H., Roach, B.J., & Ford, J.M. (2011) Relationships between pre-stimulus gamma power and subsequent P300 and reaction time breakdown in schizophrenia. Int. J. Psychophysiol., 79, 16–24. 
Romei, V., Gross, J., & Thut, G. (2010) On the role of prestimulus alpha rhythms over occipito-parietal areas in visual input regulation: correlation or causation? J. Neurosci., 30, 8692–8697. 
Todorovic, A., van Ede, F., Maris, E., & de Lange, F.P. (2011) Prior expectation mediates neural adaptation to repeated sounds in the auditory cortex: an MEG study. J. Neurosci., 31, 9118–9123. 
van Dijk, H., Schoffelen, J.M., Oostenveld, R., & Jensen, O. (2008) Prestimulus oscillatory activity in the alpha band predicts visual discrimination ability. J. Neurosci., 28, 1816–1823. 
Womelsdorf, T., Fries, P., Mitra, P.P., & Desimone, R. (2006) Gamma-band synchronization in visual cortex predicts speed of change detection. Nature, 439, 733–736.

2^nd^ Editorial Decision	01/11/18

_____________________________________________________________________________________

The relationship between the temporal structure of magnetoencephalography recorded brain activity and capacity to form discrete auditory representations 

Dear Dr. Allen, 

We are happy to provisionally accept your paper pending some very minor revisions - see reviewer comments below. Once these have been executed, we will move immediately to production. 

When revising the manuscript, please embolden or underline major changes to the text so they are easily identifiable and DO NOT leave 'track change' formatting marks in your paper.   

Please ensure that you provide a text and a figure file for the Graphical Abstract (as detailed in the instructions below). We will also need a full conflict of interest statement for publishing. When carrying out your revisions please refer to the checklist below and visit the EJN author guidelines at www.ejneuroscience.org 

When finalized, please upload your complete revised manuscript onto the website, as a Word file (.doc, or .docx).  Please also ensure that a complete set of tables and figures is included as separate files, even if these have not changed from the originals.  At this stage it is necessary to provide high resolution figures.  Please see important instructions below. 

Please go into https://mc.manuscriptcentral.com/ejn - Author Centre - manuscripts with decisions where you will find a 'create a revision' link under 'actions'. We ask that you please indicate the way in which you have responded to the points raised by the Editors and Reviewers in a letter. Please upload this response letter as a separate Word (.doc or PDF) file using the file designation "Authors' Response to Reviewers" when uploading your manuscript files.  Please DO NOT submit your revised manuscript as a new one.  Also, please note that only the  Author who submitted the original version of the manuscript should submit a revised version. 

If you are able to respond fully to the points raised, we would be pleased to receive a revision of your paper within 30 days. 

Thank you for submitting your work to EJN. 

Kind regards, 

John Foxe & Paul Bolam 
co-Editors in Chief, EJN 


Reviews: 

Reviewer: 2 

Comments to the Author 
This study examined the relationship between the temporal structure of neural oscillations and the capacity to form discrete auditory representations. An MEG protocol was pre-registered and conducted accordingly. The first analysis demonstrated that capacity to form auditory representations correlated with the prevalence and duration of activity in auditory cortex. The second analysis showed that auditory cortical oscillations entrained to stimuli over a broad range of frequencies. The third found that activity in the gamma band predicted successful representation. These findings were interpreted as evidence that a low-level function of cortical oscillations is to set conditions for representational neuronal assemblies in a frequency non-specific manner, and that this likely impacts the temporal dynamics of experience. 
The revised manuscript is a much improved report of the study under consideration. The authors have made a commendable effort to address all my concerns as well as the comments made by the other reviewer. In particular, several additional exploratory analyses have been included as supplementary information and a considerable effort has been made to include additional detail regarding background literature and critical methodological details, as well as improved data visualization. As a result, the revised article is a more transparent and the overall message is clearer. 
The only minor comments I have are: 
1. That the authors include the explanation given to Reviewer 1’s comment #4 in the discussion or elsewhere. 
2. Use the terms gamma and alpha instead of symbols in the abstract for readers that may be unfamiliar with them. Could include symbols in parentheses. 
I thank the authors for their efforts and have no further comments.

_____________________________________________________________________________________

Authors’ Response	02 /11/18

_____________________________________________________________________________________

Dear Editor,

I would like to thank you and the reviewers for the helpful input and care taken. I have made the adjustments as requested by the reviewer. Specifically, my response to reviewer 1 comment 4 has been integrated into the discussion and the words “alpha” and “gamma” have been added to the abstract. I have also included a graphical abstract as requested. I hope you find the submitted manuscript acceptable for publication.

Best wishes,

Chris

Dr Christopher Allen

Cardiff University Brain Research Imaging Centre (CUBRIC)

School of Psychology

Cardiff

CF24 4HQ

UK

Tel: +44(0)29 2068 8793

Email: [allencp@cardiff.ac.uk](mailto:allencp@cardiff.ac.uk)
